# Overlap‐Kernel EPI: Estimating MRI Shot‐to‐Shot Phase Variations by Shifted‐Kernel Extraction From Overlap Regions at Arbitrary k‐Space Locations

**DOI:** 10.1002/mrm.70196

**Published:** 2025-12-15

**Authors:** Rui Tian, Martin Uecker, Maxim Zaitsev, Klaus Scheffler

**Affiliations:** ^1^ High‐Field MR Center Max Planck Institute for Biological Cybernetics Tübingen Germany; ^2^ Institute of Biomedical Imaging Graz University of Technology Graz Austria; ^3^ BioTechMed‐Graz Graz Austria; ^4^ Division of Medical Physics, Department of Diagnostic and Interventional Radiology, University Medical Center Freiburg, Faculty of Medicine University of Freiburg Freiburg Germany; ^5^ Department for Biomedical Magnetic Resonance University of Tübingen Tübingen Germany

**Keywords:** diffusion, ESPIRiT, GRAPPA, multi‐shot EPI, self‐navigation, shot‐to‐shot phase variations

## Abstract

**Purpose:**

In multi‐shot EPI, shot‐dependent phase fluctuations can introduce ghost artifacts, undermining advantages for enhancing resolution or reducing distortion, particularly in diffusion scans. Here, a novel self‐navigation strategy based on shift‐invariant kernel extraction is proposed, enabling robust estimation of phase inconsistencies from overlap bands at any k‐space locations. It is inspired by RF sensitivity auto‐calibration in parallel imaging, and extends traditional self‐navigation trajectories beyond oversampled k‐space center.

**Theory and Methods:**

Our shot‐to‐shot correction method only requires shot‐to‐shot sampling trajectories to slightly overlap at arbitrary k‐space locations. Such k‐space overlaps can be defined as shot‐dependent auto‐calibrating‐signal regions, where a GRAPPA/ESPIRiT‐type operation can be applied to explicitly extract shot‐to‐shot signal variation maps in one step. This inherits GRAPPA/ESPIRiT's robustness, and offers much greater flexibility in designing self‐navigated multi‐shot sequences.

**Results:**

Our kernel extraction techniques in four self‐navigated 2D SE‐EPI and GE‐EPI sequences are successfully demonstrated, as well as minor applications in extracting maps from navigator data and *B*_0_ maps. Given sufficient SNR, self‐navigation EPI can reach identical/better reconstruction quality compared to navigator corrections, applicable to various trajectories. Additionally, it is particularly suitable for the two‐shot and four‐shot mosaic EPI, where stronger shot‐dependent eddy currents can also be removed, and the self‐navigated ACS has higher SNR than the refocused navigator echoes.

**Conclusion:**

This paper rigorously analyzes subspace algorithms for phase‐stabilized multi‐shot MRI, significantly broadens the self‐navigation concept, and provides a more complete picture of the connections between signal fluctuations calibration in EPI and RF sensitivity calibration in parallel imaging.

## 1 Introduction

In MRI, multiple shots of k‐space [1, 2, 3] acquisition can enhance spatial resolution and reduce geometric distortion [4]. This, however, can be undermined by the notorious shot‐dependent signal instability primarily manifested as spatially varying phase fluctuations [5, 6], ending up with ghost artifacts. This problem mostly originates from motions (e.g., respiration, heartbeat, mechanical vibration) and system imperfections (e.g., shot‐dependent eddy currents), and can become prominent in diffusion‐weighted [5] and long gradient‐echo [6] scans. Numerous correction methods [7, 8, 9, 10, 11, 12, 13, 14, 15, 16, 17, 18, 19, 20, 21, 22, 23, 24, 25, 26] have been developed to mitigate the shot‐to‐shot phase variations. Nevertheless, inherent limitations persist. Among these methods, self‐navigation [12, 14, 21, 22, 23, 24] (i.e., phase navigation from imaging data itself) offers an elegant solution, though not yet universally applicable (e.g., unsuitable for mosaic EPI segmentations [27]).

The conventional self‐navigated trajectories [14, 21, 22] can directly monitor shot‐dependent phase variations, by oversampling a central k‐space segment in each shot. However, this technique is only limited to specific trajectories and may not achieve optimal k‐space sampling. To the best of our knowledge, existing self‐navigated trajectory approaches [28] that can remove diffusion phase fluctuations (note, not translational/rotational motion corrections [29, 30, 31, 32, 33, 34, 35, 36]) generally assume that, spatially resolved shot‐to‐shot nonlinear phase maps can only be directly obtained from the k‐space central segments, while high‐frequency segments centered at the periphery of k‐space are considered insufficient for this purpose.

For multi‐shot phase‐interleaved EPI, self‐navigation can also be achieved by either estimating phase maps from intermediate parallel imaging (PI) reconstruction (e.g., inverse reconstruction method [19], and MUSE [23, 37]), or reducing shot‐inconsistency between k‐space lines or image‐space pixels via post‐processing (e.g., low‐rank matrix theory [24, 25, 26, 38, 39], e.g., MUSSEL [24] and LLR [26]/NLLR [40]). However, these methods face similar limitations: each shot should avoid excessive undersampling beyond the parallel imaging acceleration limit for recovering a full image from that shot alone, so that the combined coverage of multiple RF receivers in that shot still provides a nearly complete k‐space especially around the center, thereby avoiding errors in the shot‐to‐shot corrections. This inherently restricts the original PI acceleration benefits when considering all shots together. Additionally, iterative low‐rank regularizations may incur substantially increased computational complexity, or difficulties in interpreting converged results (e.g., residue blurring), especially for highly undersampled k‐space with strong diffusion‐weighting.

For sequences currently incompatible with the above‐mentioned self‐navigation techniques, such as readout‐segmented mosaic EPI [17, 18, 20, 41] (e.g., RESOLVE [42] in Siemens), navigator echoes can be acquired in separate scans alongside imaging echoes to estimate shot‐to‐shot phase fluctuations [7, 8, 9, 10, 11, 13, 15, 16, 17, 18, 20]. Unfortunately, the navigators prolong the total sampling duration, may not perfectly capture imaging echoes' phase (e.g., due to motions, eddy currents), could suffer from T_2_ or T_2_* signal decay, and increase SAR if acquired with additional refocusing RF pulses. These disadvantages may be more pronounced in ultrahigh field (7T or above) with shorter transverse magnetization lifetimes.

For multi‐shot mosaic EPI, there is also a magnitude‐scan strategy based on phaseless encoding MRI [43, 44, 45, 46] without need of phase navigation, which reconstructs a high‐resolution k‐space in “chunks,” from several “low‐resolution” scans with high‐frequency signals mixed into the k‐space center [44, 46, 47, 48]. The shot‐dependent k‐space aliasing can be introduced by subpixel tagging modulations prior to imaging acquisition, and resolved by tagging shifts, which inherits elements of super‐resolution microscopy [49, 50, 51, 52, 53, 54]. In this way, only the magnitude of the “low‐resolution” images is needed for super‐resolution reconstruction, removing sensitivity to inter‐shot phase fluctuations. Its main drawback is the inevitable SNR loss caused by the modulation of longitudinal magnetization, which suppresses signals from different subpixel compartments and may be unfavorable in scans using strong diffusion‐weighting with inherently low SNR.

Given these limitations, we re‐evaluate the long‐standing idea of self‐navigation MRI [14, 19, 21, 22, 23, 24, 26], and propose a novel strategy to directly extract shot‐to‐shot signal variations from imaging data in a single step, extended from GRAPPA [55] and ESPIRiT's [56] theory. It only requires k‐space region in separate shots to slightly overlap at arbitrary k‐space locations, instead of the overlap area being confined to the k‐space center [14, 21, 22]. Thereafter, a GRAPPA‐/ESPIRiT‐type operation can be applied to overlap areas to explicitly extract phase fluctuation kernels/maps that are later incorporated into the final image reconstruction. This fundamentally broadens the scope of self‐navigation and enables much greater flexibility in multi‐shot sequence design [27]. So far, non‐central k‐space overlaps have not been considered in self‐navigated trajectory designs [14, 21, 22, 28, 57] for phase extraction/correction in multi‐shot diffusion‐weighted scans yet.

Essentially, our approach is grounded in Fourier theory. The shot‐to‐shot phase variations, as the image‐space phase maps multiplied by the object's spin distribution, can be seen as k‐space kernels convolved with the original k‐space data, which are shift‐variant along shot dimension, but shift‐invariant across k‐space locations and RF receiver channels. This signal modeling is also central to the low‐rank matrix completion MRI techniques (e.g., MUSSEL [24]), and was earlier applied in calibrationless PI reconstruction [38, 58] encoded by GRAPPA [55] kernels (shift‐variant along receiver channels, shift‐invariant across k‐space locations). Here, a key insight of our work is, given the phase fluctuation kernels convolve with data in the entire k‐space rather than merely the center [24], similar autocalibrating‐signal (ACS) regions, however, between shots, can be obtained by only slightly overlapping shot‐dependent sampling trajectories at arbitrary k‐space locations. The resultant explicit phase kernels/maps extractions might also inherit the robustness of GRAPPA/ESPIRiT due to averaging effects of kernel shifts. This study is inspired by 2D phaseless encoding [46], where non‐central overlap segments [27] were used to estimate inter‐segment phase inconsistency, that was assumed as constant across image‐space. It is also inspired by the auto‐calibration technique [59] for linear and nonlinear gradient encoding [60], where even much stronger continuous phase modulation (rather than modulations only between shots) can be robustly self‐estimated via shifted kernel extraction in k‐space.

In this paper, we demonstrate the broad application of our proposed strategy termed “overlap‐kernel EPI” [61] (i.e., obtaining kernels from arbitrary k‐space overlaps), in self‐navigation using four distinct 2D EPI trajectories, and its utility to also serve as a simple and practical tool for filtering navigator and magnetic field maps [60, 62]. To emphasize the generality of shifted kernels [24, 38, 39, 55, 56, 59, 60, 63, 64, 65, 66] in order to stimulate future follow‐up work, we encapsulate our method within the generalized k‐space interpolation framework [67] and ESPIRiT theory [56], to mathematically bridge kernel‐based, explicit auto‐calibration of RF receivers and shot‐to‐shot phase variations. Furthermore, coefficient‐of‐variation (CV) maps and kernel subspace are inspected to examine the kernel/map extraction quality for the proposed algorithms. In vivo EPI scans conducted in standard and high‐performance‐gradient 3T scanners were performed to validate our theory. An early account of this work was published in conference abstracts [68, 69].

## 2 Theory

### 2.1 Four Multi‐Shot EPI Sequences, With Optional 2D Navigators For Comparison

Four multi‐shot EPI sequences [27] implemented in Pulseq [70] were used to evaluate our approach to estimate inter‐shot phase fluctuations, including mosaic (readout‐ and/or phase‐segmented) and phase‐interleaved EPI, with an option of additional 2D navigator scans for comparison. To explicitly extract phase fluctuation maps/kernels from data, at least a small (typically about 15 pixels along the shorter‐axis) k‐space overlap region between shots was required as shot‐dependent ACS regions for constructing a calibration matrix [55, 56], which needed merely subtle adjustments in pulse sequence.

**FIGURE 1:**
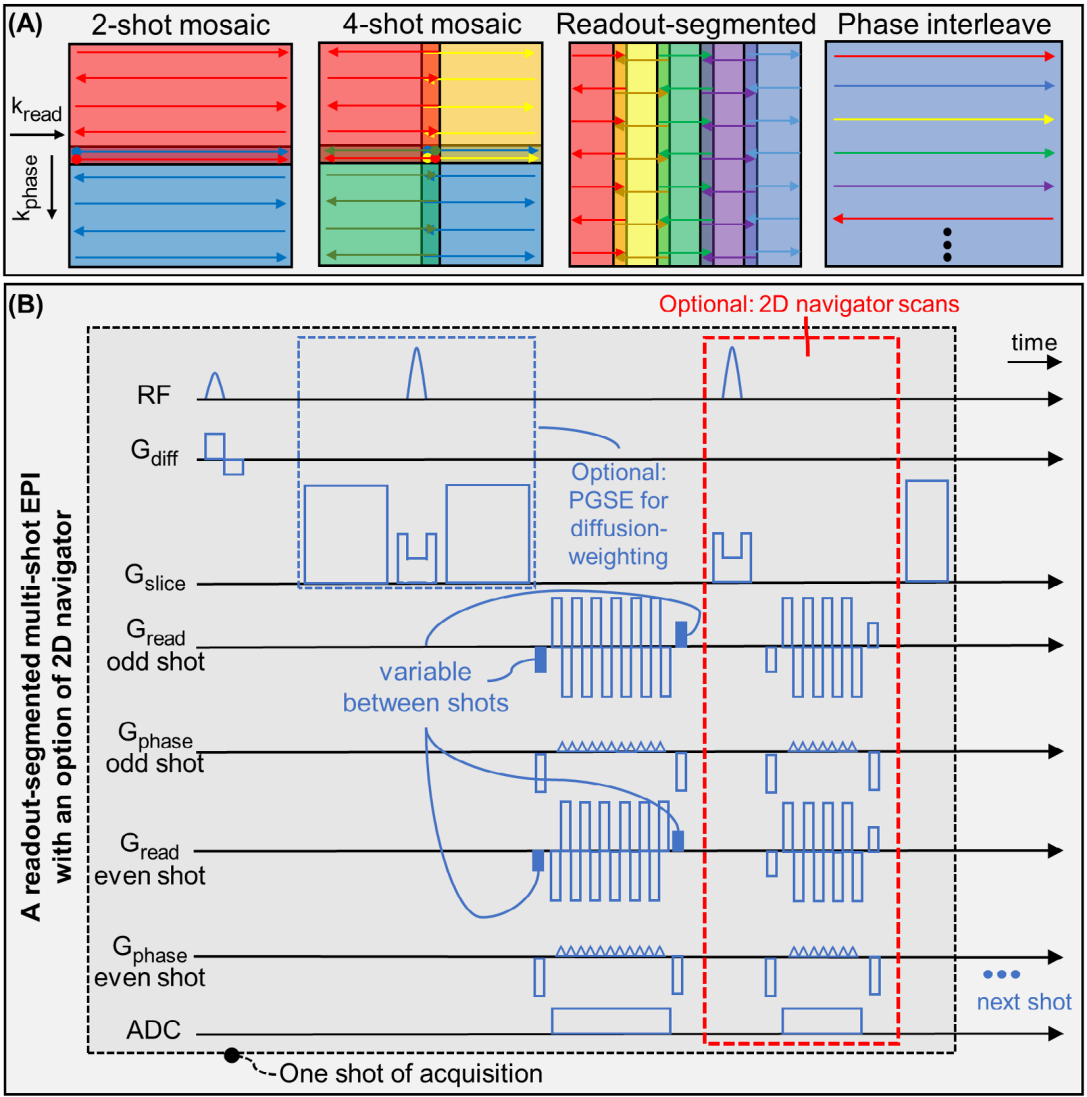
The k‐space sampling strategy and sequence. (A) The k‐space trajectory of the four EPI sequences: two‐shot mosaic EPI, four‐shot mosaic EPI, five‐shot readout‐segmented mosaic EPI, and five‐shot phase‐interleaved EPI. In the two‐shot and four‐shot mosaic EPI, the trajectories employ center‐out directions and start from the solid circles in the illustration, to minimize differences in T_2_ or T_2_* decay in overlapped regions. The density of the trajectories is drawn for illustration purposes only, and cannot reflect the realistic Nyquist sampling. In this paper, the readout‐segmented scheme was also adapted for three‐shot and seven‐shot, and the phase‐interleaved scheme was also adapted for two‐shot, three‐shot, four‐shot, and six‐shot. (B) A multi‐shot EPI sequence with optional PGSE excitation, and optional 2D navigator acquisitions. It is an example of readout‐segmented mosaic EPI, but was modified for two‐shot mosaic EPI, four‐shot mosaic EPI, and phase‐interleaved EPI. In readout‐segmented trajectory, the readout gradient polarity is flipped between odd and even shots. The readout gradient phase blips (solid blue) are adjusted between shots to sample different k‐space locations. The PGSE block used for diffusion preparation can be replaced by gradient echo excitation. The areas of gradient waveforms are drawn for illustration purposes only, and cannot exactly reflect the actual experiments.

In Figure 1, the k‐space sampling trajectories for the four EPI in‐plane segmentation schemes are presented. Additionally, a multi‐shot readout‐segmented mosaic EPI (rs‐EPI) sequence is shown as an example, with consecutive shots overlapped by, for example, 6–27 pixels along readout axis (2%–9% overlap considering a full pixel‐size of 311 pixels), and hundreds of pixels overlapped along phase‐encoding axis. Typically, traditional rs‐EPI (e.g., RESOLVE [18, 20, 42]) has only a few (e.g., 2–6) overlapped pixels along readout to avoid k‐space holes, although acquiring several more readout pixels isn't always time‐consuming and may better utilize the gradient's amplitude. Moreover, the readout gradient polarity between overlapped k‐space segments is alternated between shots, to minimize difference in transverse spin relaxation and signal dephasing, for ensuring the shot‐dependent signal differences in the overlapped k‐space originate from inter‐shot phase fluctuations only. Multiple RF receiver channels, if available (e.g., after GRAPPA reconstruction [55]), can be used jointly for phase errors estimation, as the phase fluctuation kernels are shift‐invariant across RF channels, unlike the GRAPPA kernels being unique to each receiver.

Another two multi‐shot mosaic [27] EPI sequences (center‐out, short‐TE) were designed similarly with small overlap regions, minimized differences in signal decay/dephasing between overlapped trajectories, and also equipped with an optional 2D navigator. A phase‐interleaved EPI sequence with optional navigator was also implemented for testing MUSE, while our kernel extraction approach can replace the original total‐variation image‐space phase smoothing and improve the phase map quality estimated from intermediate PI reconstruction for each shot [23]. This is similar to JULEP [71] but with only single‐step direct phase map extraction, rather than MUSSEL (iterative k‐space denoising) followed by MUSE (image‐space phase‐smoothing).

### 2.2 Phase Fluctuation Kernels Meet GRAPPA from a generalized k‐space interpolation perspective

We describe shot‐to‐shot phase fluctuations using similar notations in a reproducing‐kernel‐Hilbert‐space (RKHS) sampling theory [67] to stress a unified framework with the well‐established PI. This theory [67] universally characterizes the k‐space signal interpolations, as a concept widely used in re‐gridding [72, 73, 74, 75, 76, 77]. It can yield effective k‐space coverage and noise amplification maps for arbitrary spatial encoding functions (i.e., either maps or kernels), such as PI [55] with distinct sampling patterns (e.g., CAIPI [78], Poisson‐Disc [79]), nonlinear gradient encoding [59, 60]. While standard GRAPPA theory remains sufficient for understanding auto‐calibration of diffusion phase kernels, future work may exploit the cardinal and power function maps, unique to the RKHS formalism, to evaluate and understand phase fluctuation encoding efficiency [66] directly in k‐space.

In overlapped data segments at any k‐space locations, the time‐domain samples in shots can be written as

(1) $$f^{∥}=\mathrm{FP}m,$$

where $f^{∥}$ is k‐space signal acquired from shot number 1 to N (N≥2), F denotes Fourier transform operator, phase fluctuations can be represented by matrix $P={\left[P_{1}\;P_{2}\cdots P_{N}\right]}^{T}$ as a vertically concatenated stack of diagonal matrices $P_{i}$ with diagonal elements corresponding to the relative phase map values in each shot, m is the object signal representation seen by RF receivers.

From Fourier theory, the multiplication of phase fluctuation maps with spin distribution can be interpreted as convolution with phase fluctuation kernels in k‐space [24, 67]. Namely, within inter‐shot overlap region C seen by all RF receivers, a neighborhood of k‐space (t) data in shot (i) can be multiplied with a kernel (t,i) and summed up to approximate a sample point $\dot{t}$ in another shot j:

(2) $$f_{j}^{∥}\left(\dot{t}\right)=\sum_{t,i}f_{i}^{∥}\left(t\right)u_{j}^{t,i}\left(\dot{t}\right),$$

with $t,\dot{t}\in C$.

$f_{i}^{∥}\left(t\right)$ and $f_{j}^{∥}\left(\dot{t}\right)$ are discrete MRI samples interpolated by phase fluctuation kernel $u_{j}^{t,i}\left(\dot{t}\right)$, which is called cardinal function in the RKHS‐MRI theory to generally represent interpolation weights in k‐space for arbitrary spatial encoding functions [55, 59, 60, 78]. The notations t and $\dot{t}$ indicate time‐domain nature for k‐space. In our implementations, EPI readout oversampling is removed in this interpolation model for simplicity, without loss of generality. Note, continuous gradient errors (e.g., eddy currents) within each shot can disrupt this model for shot‐to‐shot signal variations, and may requires additional calibrations.

**FIGURE 2:**
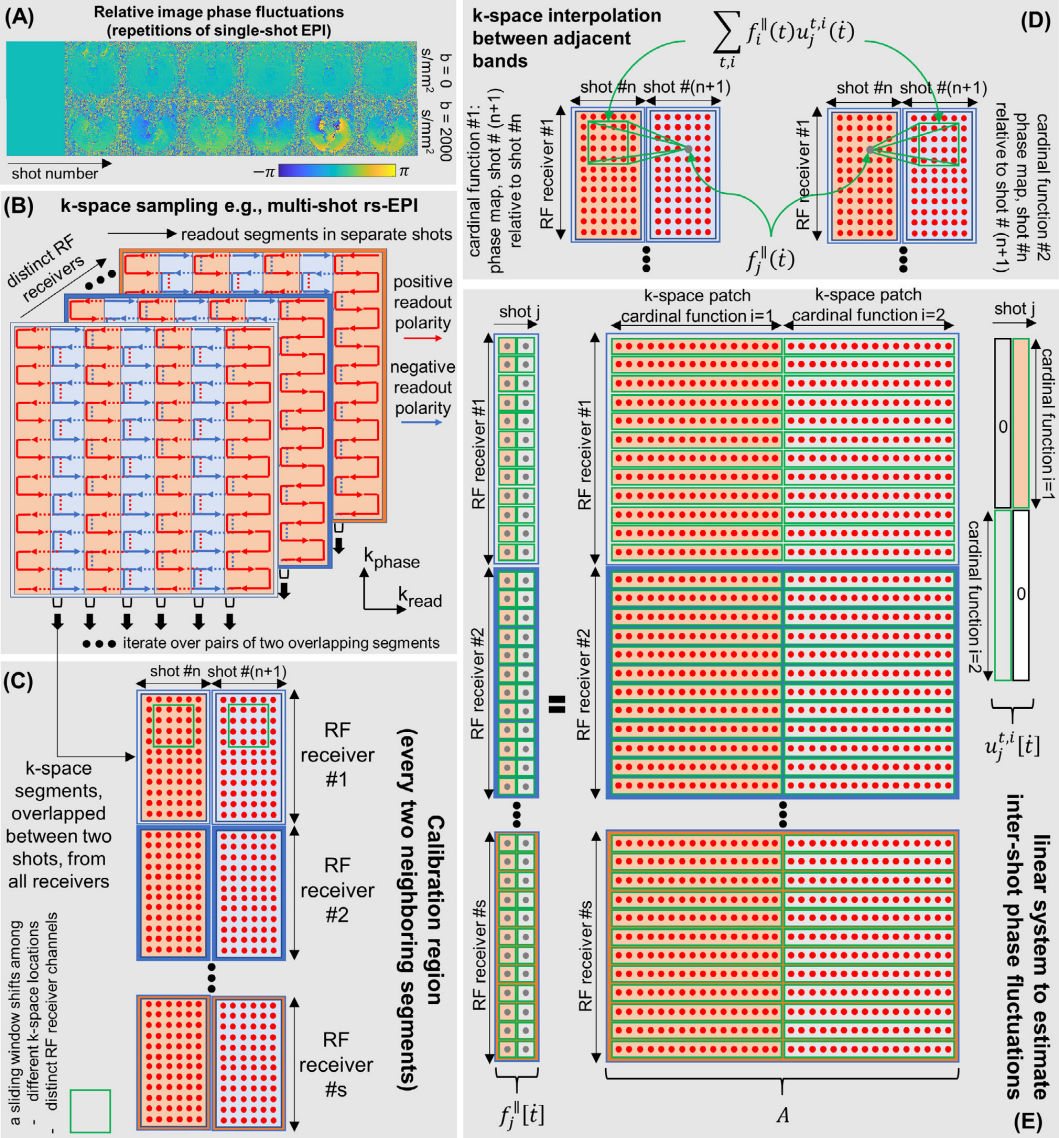
Signal processing perspective for k‐space diffusion kernel interpolations. (A) The target unknown—phase variations are demonstrated in image‐space. Their Fourier transform representations are the diffusion phase kernels. A single‐shot EPI sequence with and without diffusion‐weighting was repeated to yield these phase maps. More examples for higher *b*‐values are in Figure S7. (B) As an example, k‐space sampling trajectory for readout‐segmented EPI is shown, to illustrate the origin of the proposed shot‐dependent ACS regions (overlapped segments), for shot‐to‐shot phase extractions. (C) The calibration region constructed from every two neighboring and overlapped k‐space segments. The interpolation kernel is shift‐invariant across k‐space locations and RF receiver channels, but shift‐variant across shot numbers. (D) The k‐space interpolation relationships in the calibration region, where a neighborhood from one shot is used to interpolate a single k‐space point at a similar location in another shot. (E) The linear system representation. This system involves two cardinal functions, with the width determined by the sliding window size. In practice, only one cardinal function needs to be solved to estimate a relative phase fluctuation map between two shots, for interpolation in one direction. But both cardinal functions are shown here for a complete and general mathematical representation. Theoretically, these unknown kernels can be solved using different algorithms, e.g., pseudo‐inverse, tSVD, LSQR, or neural‐network based methods.

The kernel width usually spans several pixels (e.g., 6–20, 7 mostly used in this paper), depending on the spatial phase smoothness of the corresponding image‐space maps (e.g., Figures 2A and S7). Therefore, a calibration matrix A is constructed by applying a sliding mathematical window throughout the calibration region C, taking each k‐space block in $f_{i}^{∥}\left(t\right)$ and vectorized as a row in the matrix, as denoted by operator $R_{t}$, similar to Equation (4) [56]. The superscript (•)^*T*^ denotes nonconjugate transpose operation. In this way, a linear system with the calibration matrix can be established, linking acquired k‐space data and the unknown phase fluctuation kernels, as in Figure 2. Given operation $R_{t}$ within the calibration region C, Equation (2) can be adapted to describe this linear system:

(3) $$f_{j}^{∥}\left(\dot{t}\right)=\sum_{t,i}{\left(R_{t}f_{i}^{∥}\left(t\right)\right)}^{T}u_{j}^{t,i}\left(\dot{t}\right)=Au_{j}^{t,i}\left(\dot{t}\right),$$

Based on Equation (3), we test two approaches to estimate the phase fluctuations—directly estimating fully‐sampled k‐space kernels through a GRAPPA‐type operation [55], or calculating the corresponding image‐space phase fluctuation maps via an eigenvalue approach, as an ESPIRiT‐type operation [56]. Note, we do not directly call them “GRAPPA” or “ESPIRiT,” to stress they are not used for reconstructing multi RF‐receivers' undersampled data. Theoretically, both operations benefit from the averaging effects based on a large number of shifted kernels, inheriting the robustness of GRAPPA/ESPIRiT.

### 2.3 Phase Fluctuation Maps Meet ESPIRiT

For direct estimation of phase fluctuation kernels, the linear system in Figure 2E can be tailored for one‐way k‐space interpolation to estimate phase fluctuation of one shot relative to another. Alternatively, an eigenvalue approach can be used, and consequently, only calibration matrix A is needed to derive image‐space phase fluctuation maps, adapted from Equations (5–18) in ESPIRiT [56] for calibration of RF sensitivity maps.

To see that, the calibration matrix structure suggests the existence of null space, implying all the interpolation relationships are redundantly contained within A. Namely, the shifted k‐space neighborhoods reshaped into rows can be interpolated to approximate a data value (e.g., the neighborhood center) within that row, using the same set of cardinal functions/kernels which are shift‐invariant across rows.

(4) $$Au_{j}^{t,i}\left(\dot{t}\right)-f_{j}^{∥}\left(\dot{t}\right)=A\left(u_{j}^{t,i}\left(\dot{t}\right)-e_{\dot{t},j}\right)=0,$$

where $e_{\dot{t},j}$ is a matrix with a “1” in appropriate position that chooses the data of $f_{j}^{∥}\left(\dot{t}\right)$ in the calibration region, and “0” elsewhere.

Therefore, the calibration matrix can be decomposed using singular‐value decomposition (SVD), given the right singular‐vectors (i.e., columns of V) provide a basis for the rows of A, from which phase fluctuation kernels/maps will be extracted. The superscript ${\left(\cdot \right)}^{H}$ denotes conjugate transpose operation. Moreover, thresholding between its potential signal‐space (i.e., row space) $V_{∥}$ and noise‐space $V_{⊥}$ based on the singular‐value magnitudes allows a subspace “denoising” for the calibration data with respect to shift‐invariant kernels signals.

(5) $$A=U\sum V^{H},$$

Consequently, a subspace signal encoding perspective $Wf^{∥}=f^{∥}$ can be formulated with a operation W, representing rearranging k‐space neighborhoods into calibration matrix $R_{t}f^{∥}$, projecting onto signal‐space ($V_{∥}V_{∥}^{H}$), and reshaping back to the k‐space neighborhood ($M^{-1}\sum_{t}R_{t}^{H}$, for all shifted locations t).

(6) $$Wf^{∥}=\underset{W}{\underbrace{M^{-1}\sum_{t}R_{t}^{H}V_{∥}V_{∥}^{H}R_{t}}}f^{∥}=f^{∥},$$

where $f^{∥}$ represents general k‐space signals in Equation (1), M represents $\sum_{t}R_{t}^{H}R_{t}$ as the sample number in each k‐space neighborhood selected by $R_{t}$. Next, after inverse Fourier transforming the k‐space signals in Equation (1) with subspace projection, which can be seen as part of a image‐space operator G, the calibration consistency (6) can be expressed in image‐space:

(7) $$\mathrm{GP}m=\underset{G}{\underbrace{F^{-1}\mathrm{WF}}}Pm=Pm,$$

Similar to equation (16) in ESPIRiT [56], at every non‐zero pixel q with phase fluctuation ${\vec{p}}_{q}$, we have image‐space operator $G_{q}$ that can be written as matrix multiplication of $G_{q}^{H}G_{q}$

(8) $${\left.F^{-1}\mathrm{WF}\right|}_{q}=G_{q},$$

(9) $$G_{q}{\vec{p}}_{q}=G_{q}^{H}G_{q}{\vec{p}}_{q}={\vec{p}}_{q},$$

Such formulation transforms the problem into calculating the image‐space eigenvector of the operator $G_{q}$ with eigenvalue of 1, which is exactly the vector of phase fluctuation values. Thus, it is called an eigenvalue approach. Further, the eigenvector of $G_{q}$ can be efficiently computed by performing SVD of matrix $G_{q}^{H}$, and selecting its left singular‐vector (i.e., from $V_{G}$) with the largest singular‐value, since

(10) $$\left\{\begin{array}{l} G_{q}=U_{G}\sum_{G}V_{G}^{H}\\ G_{q}^{H}=V_{G}\sum_{G}U_{G}^{H}\\ G_{q}=G_{q}^{H}G_{q}=V_{G}\sum_{G}^{2}V_{G}^{H} \end{array}\right.$$

**FIGURE 3:**
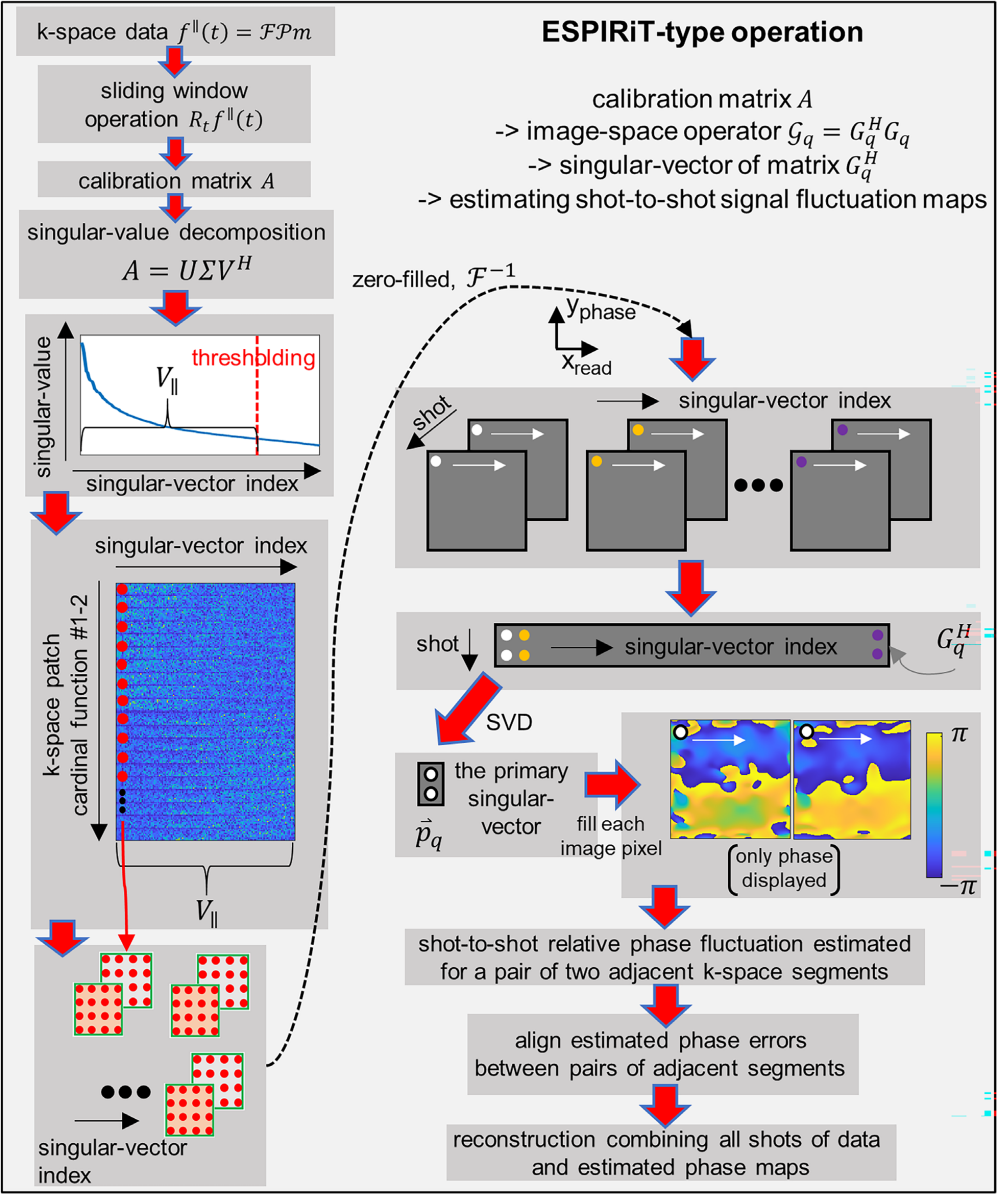
A graphical illustration of the eigenvalue approach (ESPIRiT‐type operation) for estimating shot‐to‐shot phase fluctuations. The k‐space data from the overlapping regions of two EPI segments acquired in separate shots are used to construct a calibration matrix A. Through SVD with singular value thresholding, the signal subspace $V_{∥}$ is extracted, reshaped into k‐space patches, zero‐filled, and (inverse) Fourier transform to image space. At each image‐space pixel location, a matrix $G_{q}^{H}$ across different shot numbers and singular index can be obtained. Performing another SVD on $G_{q}^{H}$, and selecting the primary singular vector corresponding to the largest singular‐value, the relative shot‐dependent signal variations at each pixel can be estimated. The phase of the estimated these variations is taken, aligned between different pairs of overlap segments, and used to correct the entire EPI segments in all shots during image reconstruction.

Namely, only $G_{q}^{H}$ is needed, obtained via Fourier transform of k‐space filters reshaped from the truncated calibration matrix subspace, corresponding to merely the left‐half part of the Equation (7) as ${\left.\left(F^{-1}M^{-1}\sum_{t}R_{t}^{H}V_{∥}\right)\right|}_{q}$. Optionally, to further reduce memory usage, $V_{G}$ can also be obtained via eigenvalue decomposition of $G_{q}^{H}G_{q}$. In practice, the primary singular‐ or eigen‐vector associated with the largest singular‐ or eigen‐value is selected to represent shot‐to‐shot signal fluctuation maps. The eigenvalues of the (normalized) operator $G_{q}=G_{q}^{H}G_{q}$ generally lie between 0 and 1, because in $F^{-1}\mathrm{WF}$, the Fourier transform is unitary and the operator W representing an average of projections has eigenvalues smaller or equal to one. In this way, the diffusion fluctuation maps can be estimated pixelwise as the eigenvectors of the operator $G_{q}$ associated with the eigenvalue of 1. These steps are graphically illustrated in Figure 3.

Note, if phase estimation takes place after combining RF receivers, it is not straightforward to have the “soft” forward model [56] in PI, since there is no encoding redundancy to estimate multiple sets of phase fluctuation maps. Additionally, the residue null‐space images [56] cannot be easily produced since EPI segments overlap only in small regions.

### 2.4 Reconstruction to Combine all Shots

Given undersampled k‐space data, PI reconstruction can be applied to at least overlap k‐space region for yielding shot‐dependent ACS. This can be called self‐navigated ACS for imaging echoes, or separate navigators' ACS if phase is extracted from 2D navigators via the kernel‐based operations. Afterwards, there are two ways for reconstructing the final image combining all shots.

For the first approach [80] (here, used for mosaic EPI), if fully‐sampled partial k‐space segments are obtained after PI (e.g., ESPIRiT/GRAPPA) reconstruction, they can be simply multiplied by the complex conjugate of estimated phase maps in the corresponding shots, and summed up into a final k‐space image, with an inverse filter [45] to remove modulation‐transfer‐function (MTF) due to the partial k‐space overlaps. Optionally, an apodization window [45] can be applied on k‐space segments to reduce PSF ringing before phase corrections, and will be eventually removed by the inverse filter also.

In the second approach [19] (here, used for phase‐interleaved EPI), the k‐space data, RF receivers sensitivity maps/kernels and estimated phase maps/kernels can be fed into a forward model to reconstruct a final image, which includes phase fluctuation encoding [66] in this multi‐shot PI reconstruction.

## 3 Methods

### 3.1 MRI Sequence

The 2D multi‐slice, multi‐shot EPI sequences with optional 2D navigators were implemented in Pulseq [70], with slight adaptations for the four different trajectories. The experiments were conducted on two commercial 3T human MRI scanners (Prisma^fit^ with max. 80 mT/m and 200 T/m/s, Cima.X with max. 200 mT/m and 200 T/m/s, Siemens Healthineers, Erlangen, Germany), including ex vivo brain phantom scans [81, 82] and in vivo brain scans (several healthy adults volunteers, in agreement with the institution's ethics policy), all using Pulseq sequences with off‐line image reconstruction. The scan protocol included pulsed‐gradient‐spin‐echo (PGSE) diffusion‐weighted (*b*‐values from 0 to 3000 s/mm^2^) or gradient‐echo (GE, TE 30 ms) EPI scans, reference ACS scans for PI reconstruction (i.e., low‐resolution GRE), EPI projection pre‐scans (i.e., phase‐encoding gradient disabled) for odd‐even‐echoes correction, and optionally, *B*_0_ mapping scans consisting of two GRE acquisitions with 3 ms difference in TE.

The in‐plane spatial resolution is denoted as “mm (readout dim.) × mm (phase‐encoding dim.)”. For sequences with a navigator acquisition, the echo time is specified as “TE imaging/TE navigator.” The FOV was 220 mm^2^, with the slice thickness of 3 mm. The volumetric one‐shot TR (one shot for acquiring all slices) was 4 s for SE‐EPI, and 1 s for GE‐EPI. The partial Fourier acquisition was disabled in two‐shot mosaic, four‐shot mosaic, two‐shot phase‐interleaved EPI and was 6/8 for all other sequences.

The scan‐dependent sequence protocols (e.g., resolution, shot, ESP, gradient, overlapped pixels, PI acceleration factors, slice number, *b*‐value, diffusion directions) are documented in Supporting Information. The ESP is defined as the time between the centers of two consecutive EPI readout lines, and the effective ESP further considers the undersampling and phase‐interleaved factors to reflect distortion levels.

### 3.2 Image Reconstruction

The images were reconstructed offline in MATLAB (CPU, 2.35GHz, 64 cores) from raw data, consisting of the following steps. Detailed implementation for Step 4 (shot‐to‐shot phase extraction) is documented in Supporting Information Section 9.

1. EPI re‐gridding based on nominal trajectory, with readout oversampling eliminated.
2. N/2 ghost corrections based on separate FID scans.
3. SENSE reconstruction [83] with pre‐whitening and ESPIRiT map [56]. Distortion correction with *B*_0_ maps was incorporated into the forward model, except for Figures 6, 7, S6, and S8. This step can be replaced by applying GRAPPA reconstruction over overlap regions only.
4. Estimation of shot‐dependent phase fluctuation maps using one of the four presented techniques in Figure 6. The computational complexity for kernel extraction is similar to GRAPPA/ESPIRiT, but with much less shot number than RF receiver number. A fast‐prototyped MATLAB implementation took below 1 s for GRAPPA‐type operation, about 1–2.5 s for ESPIRiT‐type operation, to compute 2–5 relative shot‐to‐shot phase maps of one slice, in one of the mosaic EPI sequences or 2D navigators. The fast‐prototyped ESPIRiT‐type operation took within 12 s for one‐slice‐five‐shot phase maps in phase‐interleaved EPI.
5. Combining all shots of data and phase maps, using one of the two approaches in Section 2.4.
6. Partial‐Fourier reconstruction [84]. The factor is defined as the ratio between actual‐ and fully‐sampled lines within the first half k‐space.
7. Calculation of tSNR or its inverse (coefficient‐of‐variation map, CV), or export to FSL‐DTI toolbox [85].

## 4 Results

### 4.1 Robust and Versatile Self‐Navigation for Different Trajectories

**FIGURE 4:**
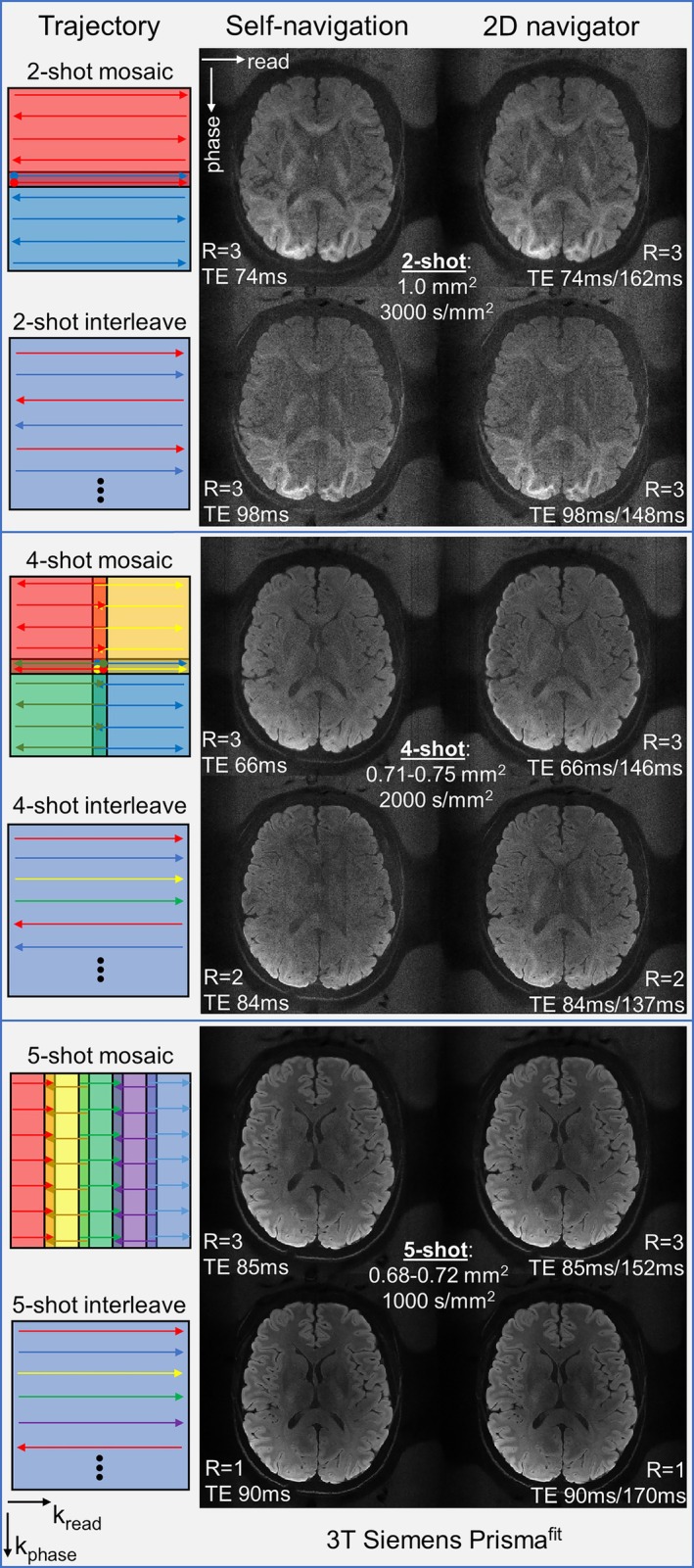
Multi‐shot EPI sequences, comparing self‐navigation and 2D navigator corrections, mosaic and phase‐interleaved (in‐plane) segmentation schemes. Here, reconstructions for self‐navigation and 2D navigator corrections are based on the same scan, equipped with both k‐space band overlap and 2D navigator acquisitions. The overlap regions are located at k‐space center, a bit away from center, or far away from center in different sequences, as in the trajectory graphs. The sum images consisting of 16 diffusion‐encoding directions are shown, with a full‐version figure with difference maps (between self‐navigation and 2D navigator corrections) and subspace thresholds in Supporting Information (Figure S2). The eigenvalue approach (ESPIRiT‐type operation) with 7 × 7 kernel size was utilized for extracting phase maps from self‐navigated ACS regions and separate navigator's ACS region. Identical shot number and similar resolution was employed to compare mosaic and phase‐interleaved schemes, where the latter can suffer from low‐quality intermediate reconstruction for each shot. The parallel imaging acceleration factor R is defined as the undersampling factor along phase‐encoding considering all shots of data relative to a full k‐space. Thus, in phase‐interleaved EPI, the k‐space undersampling factor in intermediate PI reconstruction is the multiplication of R and shot number. Typically, 2D navigator acquisition cost a few tens of milliseconds per shot. In comparison, the additional sampling time due to k‐space overlapping is negligible. More detailed sequence comparisons should be based on more considerations in acoustic resonance, PNS, distortion, gradient capabilities [86, 87, 88], wave‐encoding [89], and is beyond the scope of this paper.

In Figure 4, our shot‐to‐shot phase estimation method (here, the eigenvalue approach) enables robust and versatile self‐navigated multi‐shot EPI on a standard 3T scanner, using four trajectories—two‐shot mosaic (center‐out), four‐shot mosaic (center‐out), five‐shot readout mosaic, five‐shot phase‐interleaved (MUSE with kernel extraction). Their sum images consisting of 16 diffusion directions show no visible differences to the corresponding reconstruction corrected with separate 2D navigators. Furthermore, at matched shot number and final resolution, the two‐shot and four‐shot mosaic EPI show substantially higher SNR due to shorter TE, and improved four‐shot self‐navigation quality due to fully‐sampled (after PI), high‐SNR shot‐dependent ACS, compared with phase‐interleaved EPI. A full‐version figure with difference maps between self‐ and external‐navigation, and subspace thresholds is shown in Supporting Information (Figure S2).

Specifically, the two‐shot mosaic and phase‐interleaved EPI had effective ESP of 0.36 ms/0.18 ms, *b*‐value 3000 s/mm^2^, and no partial‐Fourier. The two‐shot mosaic sequence incurred two additional EPI readout lines (3× undersampled before PI) per shot for k‐space overlap (overlap‐size 222 × 13), corresponding to extra 5.4 ms per 2D acquisition, as negligible. However, with 24 ms TE reduction, it shows markedly clearer image structure than the phase‐interleaved EPI (central overlap 50 × 50, from intermediate PI reconstruction) for strong diffusion‐weighing, both with and without 2D navigators.

The four‐shot mosaic EPI had effective ESP of 0.23 ms, no partial‐Fourier, and increased resolution from two‐shot mosaic EPI without additional k‐space MTF due to T_2_ decay. It required three additional EPI readout lines (3× undersampled before PI) per shot for k‐space overlap (overlap‐size 13 × 155), corresponding to extra 5.6 ms per 2D acquisition, as negligible. The four‐shot phase‐interleaved EPI had effective ESP of 0.14 ms, 6/8 phase‐partial‐Fourier, and 18 ms longer TE. Given *b*‐value 2000 s/mm^2^, the image quality of four‐shot phase‐interleaved EPI degrades, especially in self‐navigation reconstruction (MUSE with kernel) that failed to estimate accurate phase maps from a central 50 × 50 overlap based on the intermediate PI (here, ESPIRiT) reconstruction. This is because, in this case, 8× undersampling per shot severely exceeds (intermediate) PI's capability for image recovery.

The five‐shot readout‐segmented EPI had effective ESP of 0.18 ms, phase‐partial‐Fourier 6/8, and overlap regions of 16 × 274. For this protocol (five‐shot, 0.72 mm^2^), the additional 16 readout‐overlapped points did not actually cause additional acquisition time, but just slightly raised the under‐utilized readout gradients to 22 mT/m and 137 T/m/s. Here, ESP cannot be further shortened because of the prohibited acoustic resonance band (Figure S3). If ignoring this prohibited band, using similarly underutilized gradient strength/slew rate, effective ESP can be further reduced to 0.15 ms. Note, in other protocols using very short ESP (e.g., below 0.4 ms, 0.13 ms effective with *R* = 3) above the prohibited acoustic band, very narrow readout‐segments (e.g., spanning 30 k‐space pixels, for lower spatial resolution) may be used, which can make such 16 overlapped points non‐negligible and require protocol‐specific re‐evaluation of feasible overlap schemes. The five‐shot phase‐interleaved EPI had effective ESP of 0.22 ms, phase‐partial‐Fourier 6/8, and overlap region of 50 × 50 from intermediate PI (here, ESPIRiT) reconstruction. Because of merely 5× undersampling per shot relative to a full k‐space, namely, higher gradient amplitude (43 mT/m, 138 T/m/s) to sample more k‐space data, five‐shot phase‐interleaved EPI finally yields identical self‐navigated image quality to the reconstruction corrected by 2D navigators.

**FIGURE 5:**
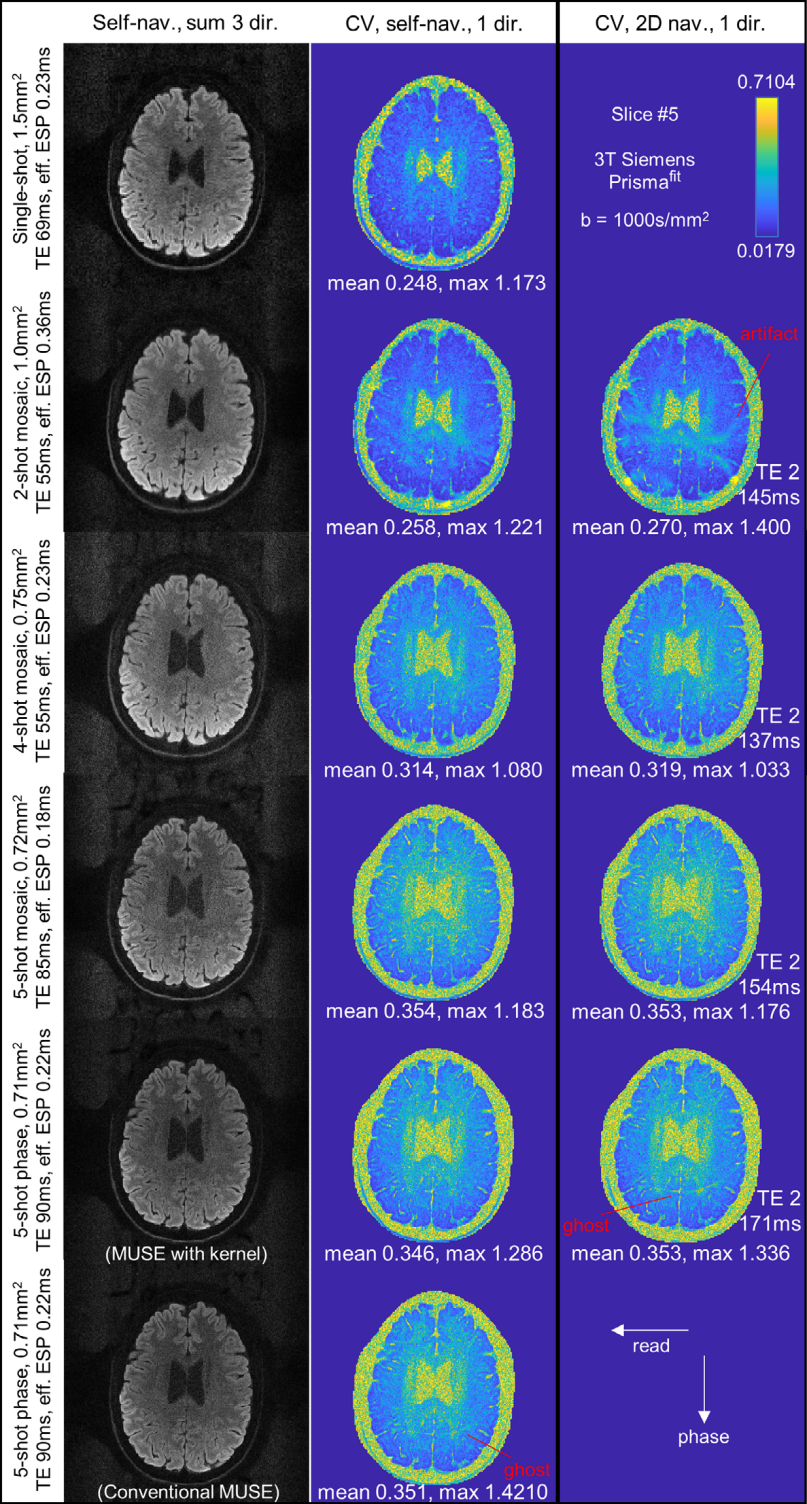
Quantitative measurements of shot‐to‐shot phase calibration errors, for the multi‐shot EPI sequences. Self‐navigated sum images consisting of 3 diffusion directions, and coefficient‐of‐variations (CV) maps for scans with 1 diffusion‐direction over 10 repetitions are shown. Different sequences—single‐shot, two‐shot mosaic, four‐shot mosaic, five‐shot readout‐segmented mosaic, five‐shot phase‐interleaved with 1000 s/mm^2^ were used. Note, the effective ESP (not ESP) are shown, as the ESP divided by k‐space undersampling (including phase‐interleaved if available) factor in each shot. Although no apparent artifacts are found in all reconstructed sum images, two‐shot and four‐shot mosaic EPI has visibly higher SNR, cross‐validated by lower CV values, compared to the five‐shot readout‐segmented and phase‐interleaved EPI. Nevertheless, the two‐shot and four‐shot mosaic EPI also remain more sensitive to *B*_0_ field offsets similar to spiral, which can cause occasional blurring in other slice positions and requires more optimized corrections for trajectory or *B*_0_ inhomogeneity, as shown in Supporting Information (e.g., Figure S10). Generally, CV maps for self‐navigation reconstruction appear lower or similar to 2D navigator corrections, indicating 2D navigators should be removed under similar *b*‐values and B_0_ inhomogeneity levels. Notably, in 2D navigator corrections for two‐shot mosaic and four‐shot phase‐interleaved EPI, as well as conventional MUSE (image‐space total‐variation phase extraction) [90], additional artifact or ghosts can be clearly observed in CV maps, indicating time‐course instability in their corresponding shot‐to‐shot phase estimations.

In Figure 5, as further quantitative measure, coefficient‐of‐variation (i.e., 1/tSNR) maps (10 repetitions, one out of three diffusion directions) demonstrate improved accuracy in our self‐navigation approaches based on explicit kernel extraction, relative to 2D navigator corrections or conventional MUSE (with total‐variation‐phase‐smoothing). Here, a 7 × 7 kernel size was used with around half subspace truncation for all kernel extraction algorithms. Particularly, in two‐shot mosaic and five‐shot phase‐interleaved EPI, CV maps for reconstruction corrected by 2D navigators or MUSE reveal additional artifacts or ghosts [90], which are minimized in our kernel‐based self‐navigation methods. The artifacts in 2D navigator corrections may partially result from the shot‐dependent eddy currents that cannot be captured by navigators at k‐space center. Additionally, the two‐shot and four‐shot mosaic EPI achieved higher SNR relative to more commonly used readout‐segmented and phase‐interleaved EPI, validated by smaller CV values.

**FIGURE 6:**
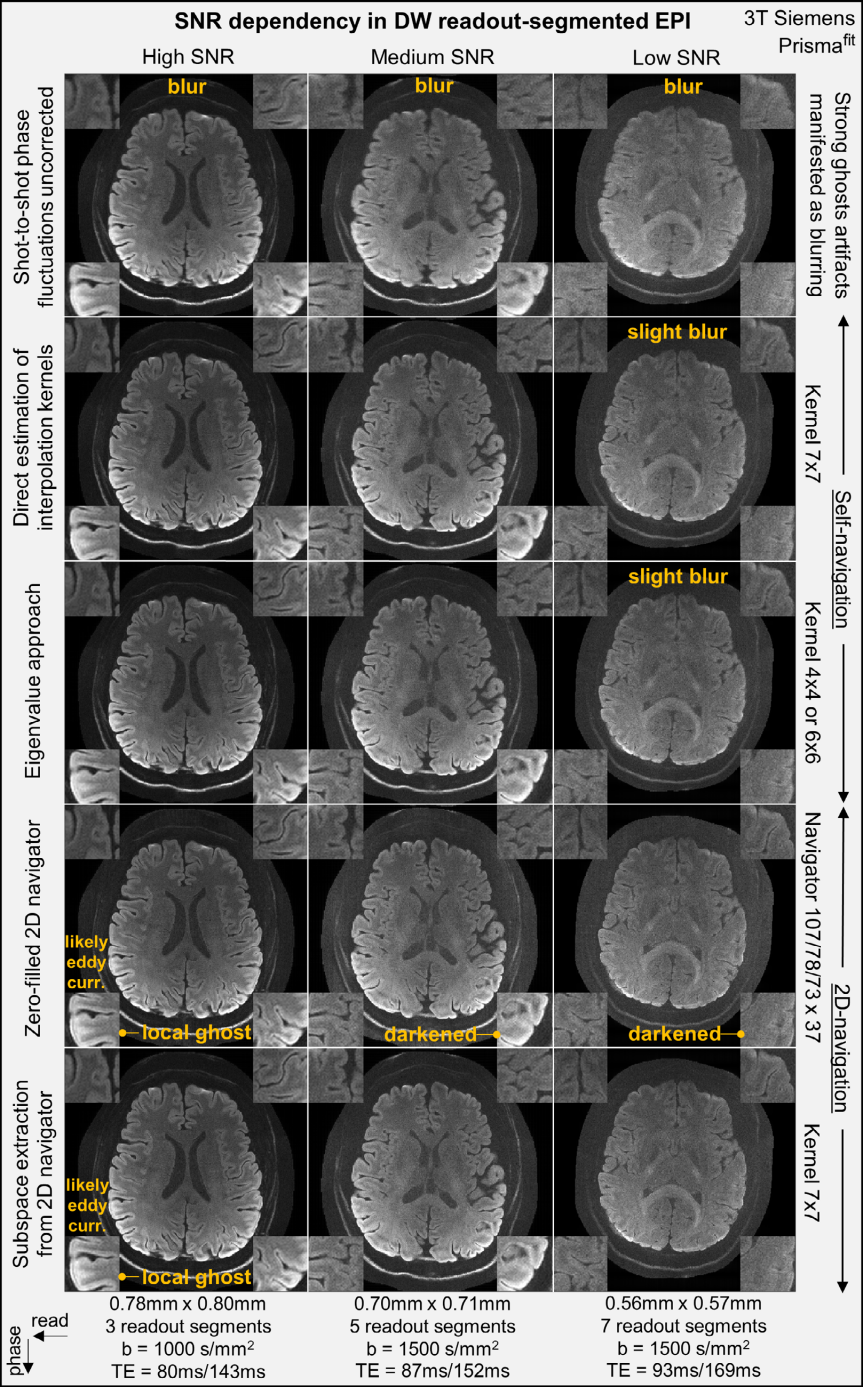
Multi‐shot readout‐segmented EPI with different methods for correcting shot‐dependent phase fluctuations. The diffusion‐weighted images are shown as the sum image for a total of 20 or 16 diffusion directions. Column 1–3: High, medium and low SNR scans with increasing resolution, more readout segments, larger diffusion‐weighted *b*‐values and longer echo time (TE imaging echoes/TE navigator echoes). Row 1–5: The five approaches without and with shot‐to‐shot phase fluctuations corrections.

Note, phase maps extraction from central k‐space data—2D navigators, phase‐interleaved EPI (i.e., MUSE with kernel extraction), and *B*_0_ field scans (Supporting Information) was also implemented via an ESPIRiT‐type operation; their comparisons with non‐kernel‐based phase extraction are provided in Figure 6 and Supporting Information (Figures S4 and S5). Moreover, shot‐dependent eddy currents (only captured by self‐navigation rather than additional navigators), subspace choices (kernel sizes and thresholds), measured diffusion kernel widths, increasing *b*‐values, quantitative multi‐shot reconstruction errors across different slices are analyzed in Supporting Information (Figures S6–S13).

### 4.2 Kernel Extraction Accuracy: SNR and Subspace Analysis

Nevertheless, the estimation accuracy of our overlap‐kernel approaches can degrade in very low‐SNR calibration regions [12]—for example, in peripheral k‐space regions for readout‐segmented sequences given strong diffusion‐weighting. Both explicit kernel extraction approaches (direct estimation of interpolation kernels, and eigenvalue approach) exhibit similar problems under these conditions, which can be much worse than the central k‐space, non‐diffusion‐weighted ACS in PI [91].

Thus, in Figure 6, we evaluate kernel extraction accuracy in terms of visual quality, using readout‐segmented EPI sequence as an example. Four different shot‐to‐shot phase estimation algorithms (Supporting Information Section 9) are compared—GRAPPA‐type operation (linear inversion using Moore‐Penrose inverse), ESPIRiT‐type operation, low‐pass‐filtered and zero‐filled 2D navigator, and kernel‐extracted 2D navigator. Multi‐shot reconstructions from high‐ to low‐SNR conditions are compared, corresponding to increasing shot number, resolution, diffusion‐weighted *b*‐values, TE, all of which can result in low‐SNR in non‐central overlapped k‐space segments. Generally, all phase fluctuation correction algorithms (Rows 2–5) provided significantly higher spatial resolution than the uncorrected scan with strong blurring (Row 1), revealing more detailed image texture.

In high‐ and medium‐SNR, both two self‐navigated reconstructions yield equal or better image quality than 2D navigator corrections (e.g., with local ghost). This is possibly because, they can also calibrate shot‐dependent eddy currents (e.g., due to shot‐dependent EPI pre‐phasers, Figure S6), which cannot be captured by navigator echoes that are always at k‐space center. However, in low‐SNR regimes, 2D navigators provided much more sufficient SNR at central k‐space regions for phase estimation, while the self‐navigated reconstructions appear slightly blurred.

Additionally, reconstructions using shot‐to‐shot navigator phases derived from kernel extraction (here, ESPIRiT‐type operation) appear more robust than simply low‐pass filtering and zero‐filling the navigator data (e.g., local darkening).

**FIGURE 7:**
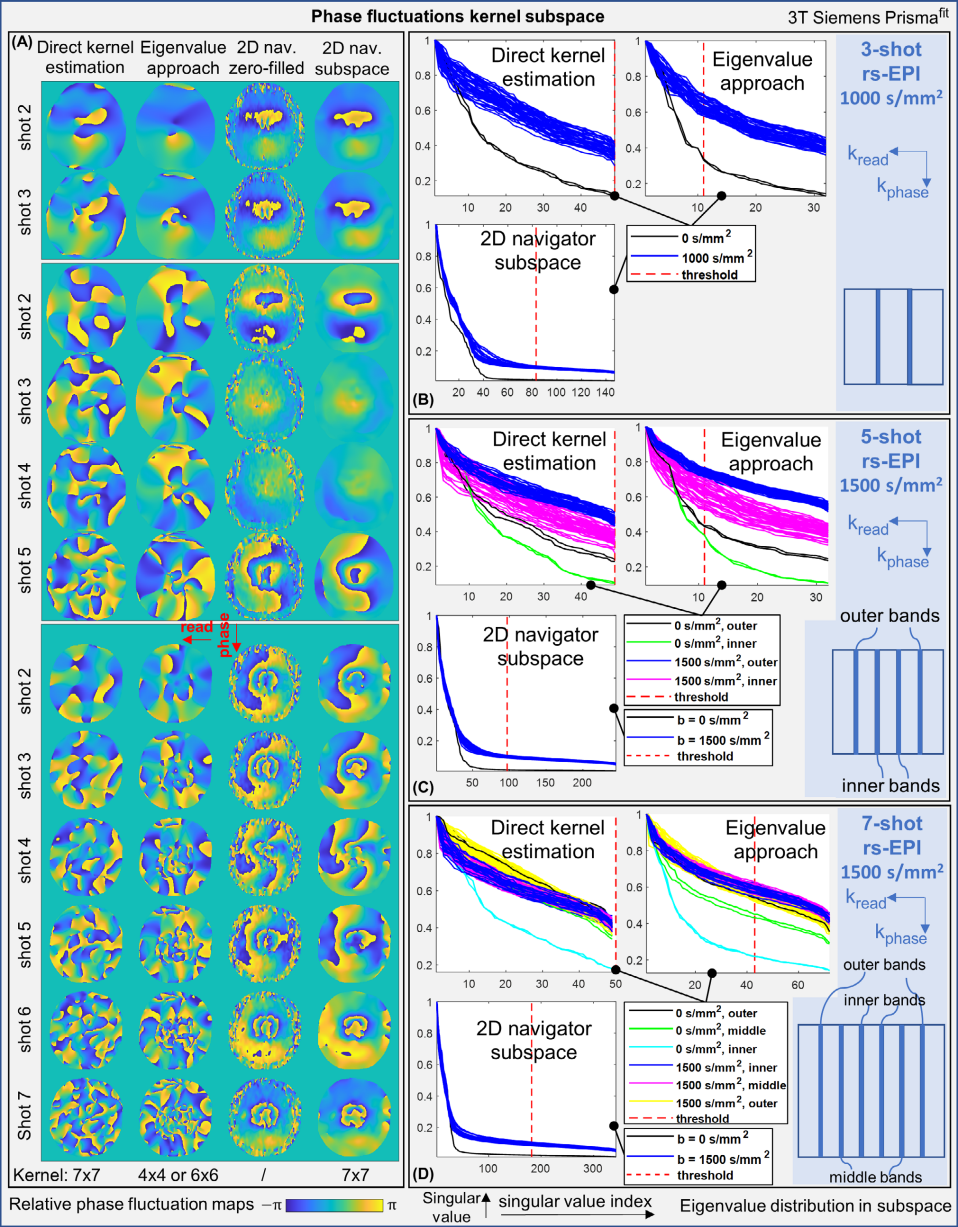
Estimated diffusion phase fluctuation maps by different methods, and the singular‐value distributions from the kernel extraction algorithms applying to the overlapped imaging data at different k‐space locations, as well as the 2D navigator data at the k‐space center. Subspace for different diffusion *b*‐values are also shown.

To further inspect and visualize the extractability of diffusion phase kernels, in Figure 7, estimated relative shot‐dependent phase maps and singular‐value distributions of kernel subspace are demonstrated for the four phase extraction algorithms in Figure 6. Due to phase wrapping, possibly caused by different kernel extraction algorithms (i.e., numerical consequences rather than physical modulations), a direct comparison between the estimated phase maps is not straightforward.

Nonetheless, a more low‐rank (or left‐concentrated/flattened) singular‐value distribution can imply that, the k‐space kernels could be represented with fewer, well‐defined singular vectors—leading to higher accuracy in extracting inter‐shot signal variations in subspace. Generally, both the direct kernel estimation and the eigenvalue approach had similar singular‐value distributions for kernel subspace, indicating their similar multi‐shot reconstruction quality (Figure 6). The low‐rank property can be substantially reduced by stronger diffusion‐weighting, or shifting the k‐space locations from inner to outer overlapped bands. Consequently, the most outer bands in the five‐shot and seven‐shot readout‐segmented EPI with 1500 s/mm^2^ exhibit the least low‐rank kernel subspace, indicating largest estimation errors. Meanwhile, as always located at central k‐space regions, 2D navigator data had consistent low‐rank kernel subspace across different *b*‐values (here, up to 1500 s/mm^2^), indicating sufficient SNR for robust shot‐to‐shot diffusion phase extraction.

### 4.3 Increased *b*‐Value Limits in High‐Performance Gradient Scanners

As in Figures 6 and 7, the phase estimation errors in low‐SNR overlapped k‐space segments—often observed in very peripheral regions under strong diffusion‐weighting—can lead to slightly blurred reconstruction compared to 2D navigator correction. Importantly, this reflects a fundamental limit of DW‐MRI signals at outer k‐space, which affects all signal processing techniques based on those regions [24], rather than a specific shortcoming unique to our kernel extraction algorithms.

**FIGURE 8:**
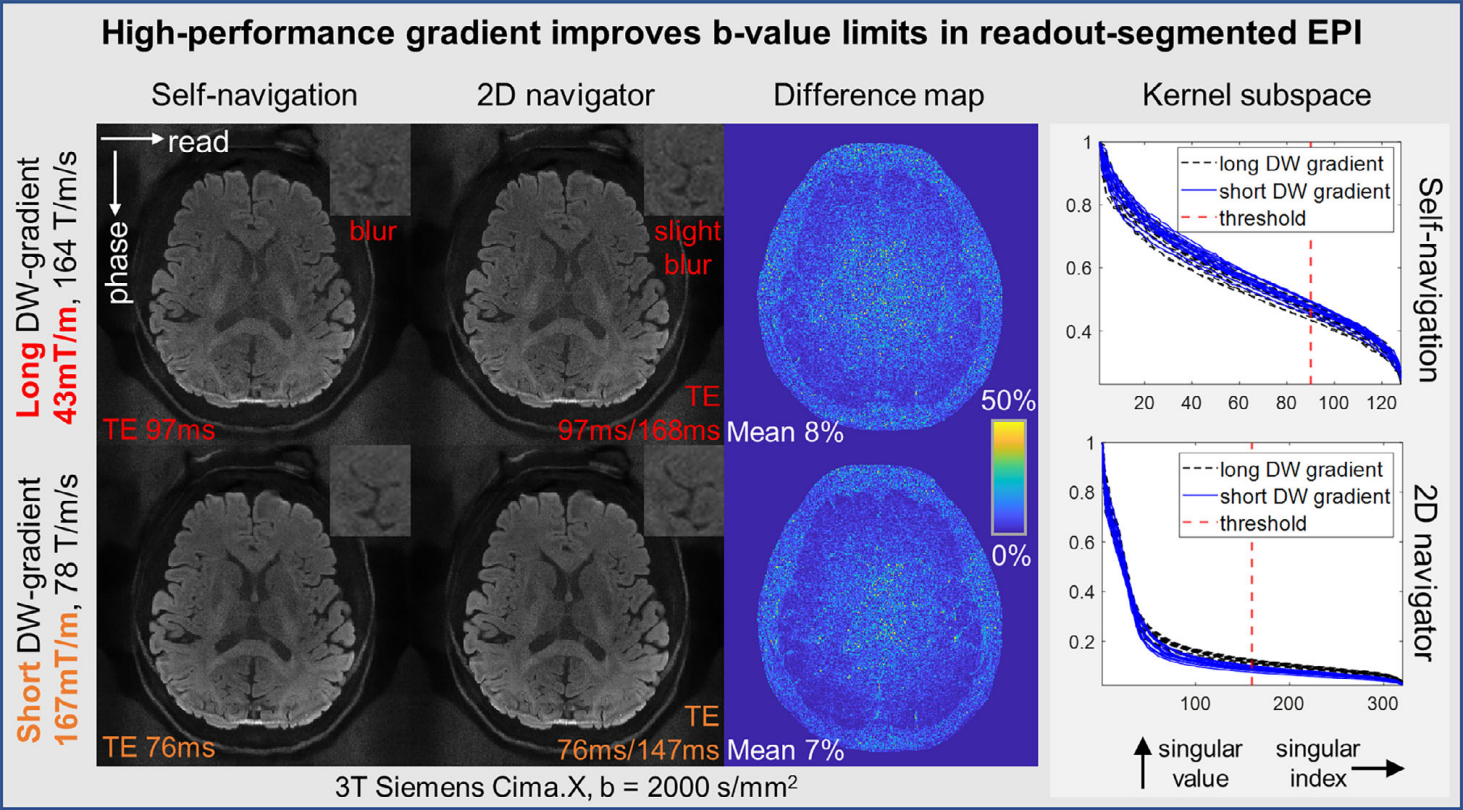
Five‐shot readout‐segmented EPI in 3T Siemens Cima.X scanner. Long (a protocol to emulate gradient‐limits of the Prisma scanner) and short (feasible only with the gradient performance of the Cima.X scanner) diffusion encoding gradients were utilized, comparing the estimation accuracy of shot‐to‐shot phase variations based on non‐central k‐space overlap regions. The eigenvalue approach (ESPIRiT‐type operation) was used with kernel size 7 × 7, for both self‐navigated ACS and navigator's ACS. In self‐navigated kernel extraction, the singular‐value distribution appears slightly more dispersed in the scan with long diffusion gradient, than the short one, which might indicate signal instability due to low‐SNR. Note that, due to the dominant cardiovascular nerve stimulation (CNS) limits by whole‐body gradient in the Cima.X scanner, the slew rate of diffusion gradients was far below the nominal hardware limit (200 T/m/s). This might be further mitigated [92] in the future to enable self‐navigation with higher *b*‐values for the widely adopted RESOLVE sequence.

Therefore, in Figure 8, we demonstrate the possibility to address this limitation in specific sequences, by employing a high‐performance gradient scanner (3T, Siemens Cima.X). A five‐shot readout‐segmented EPI with long and short diffusion‐encoding gradients was tested for *b* = 2000 s/mm^2^, with identical EPI readout train for comparison.

With a long (slow) diffusion‐encoding gradient (comparable to 3T Siemens Prisma^fit^), the self‐navigated EPI reconstruction exhibits stronger local blurring relative to the 2D navigator corrected reconstruction, as in the zoomed region.

With a short (fast) diffusion‐encoding gradient, there is no visible difference in resolution between self‐navigated and the 2D navigator‐corrected images—the zoomed region confirms equivalently resolved local image texture.

### 4.4 DTI And GE‐EPI

**FIGURE 9:**
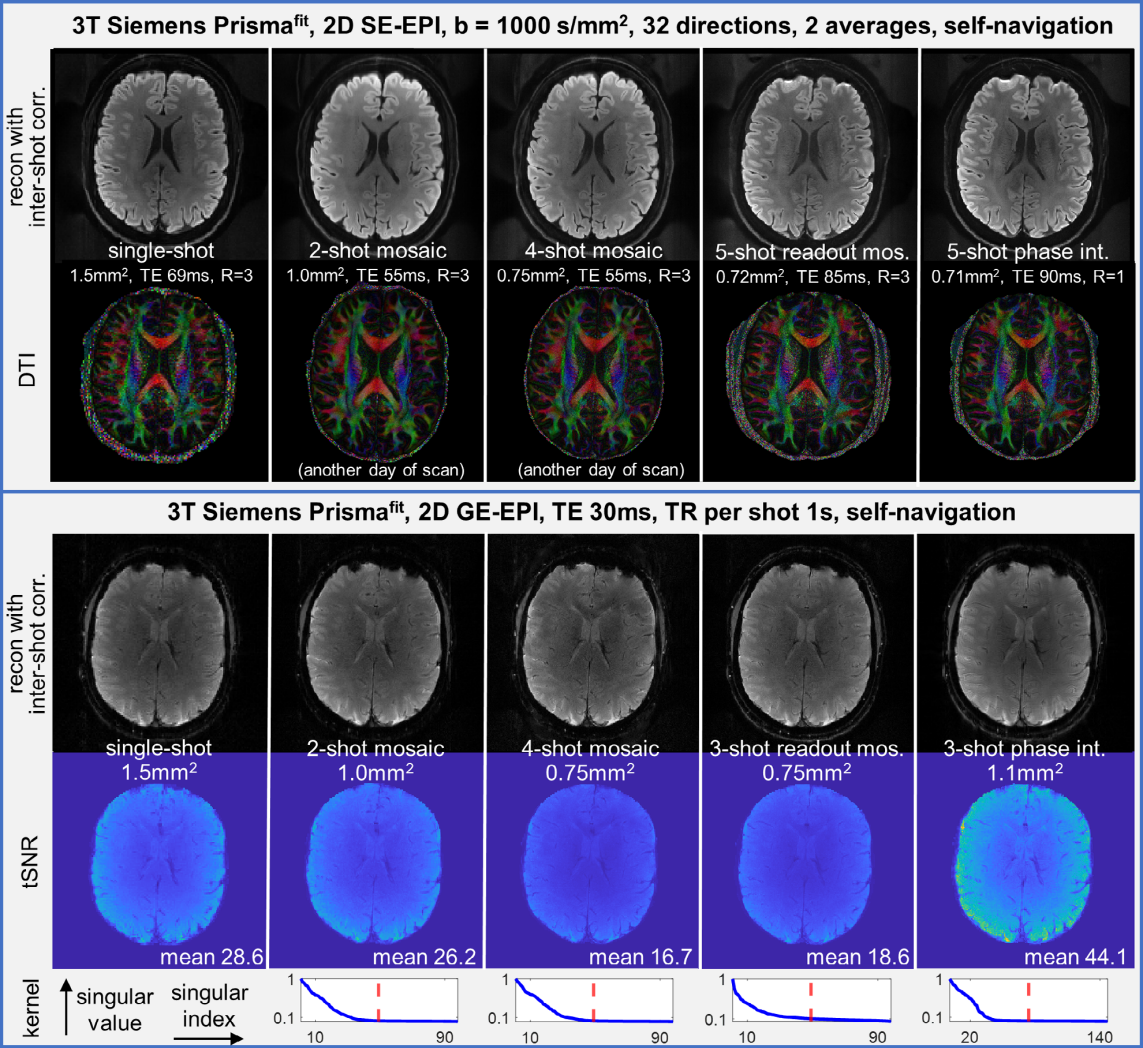
DWI, DTI, GE‐EPI images with the four self‐navigated EPI sequences. Upper: The DWI/DTI were acquired with 2D SE‐EPI with 1000 s/mm^2^, 32 diffusion directions, and 2 averages. Their ESPs are the same as the sequences in Figure 5. The 2D navigator was removed and the time duration of one‐shot‐one‐slice acquisition became shortened by at least a few tens of milliseconds, as documented in Supporting Information. The two‐shot and four‐shot mosaic diffusion scans were taken on another day of separate scan with smaller slice number and shim volume, due to sensitivity to *B*_0_ inhomogeneity. Otherwise, all other SE scans were performed with 26 slices, and all GE scans were performed with 11 slices. The two‐shot and four‐shot mosaic EPI yielded much higher SNR due to shorter TE. In GE‐EPI, the kernel extractability at non‐central k‐space regions (e.g., readout mosaic) became much more reliable than diffusion‐weighted scans due to sufficient SNR, although shot‐to‐shot phase fluctuations appear much weaker than in strong diffusion‐weighted scans at 3T. More slices positions in these diffusion‐weighted scans, as well as artifacts patterns in uncorrected multi‐shot reconstructions are shown in Supporting Information. The latter can serve as important indications for protocol selections, when specific artifacts patterns should be strictly avoided (e.g., ghosts which may replicate fake functional activations at a distant brain location due to phase‐interleaved trajectories).

Figure 9 shows the neuroimaging applications of our proposed self‐navigated sequences without 2D navigators, including diffusion‐weighted imaging, DTI (e.g., the principal diffusion direction maps), and time‐series GE‐EPI. The artifacts induced by multi‐shot phase fluctuations in the uncorrected images, which manifest themselves in both the object and background in diverse forms [12] (Figure S9), were all effectively removed by kernel extraction (here, eigenvalue approach) to allow reliable resolution enhancements. Notably, the SNR in two‐shot and four‐shot mosaic DW‐EPI were visibly higher. Furthermore, the tSNR maps for GE‐EPI confirm the absence of localized temporal instabilities due to uncorrected multi‐shot artifacts, demonstrating that these self‐navigated sequences are well‐suited for fMRI applications.

## 5 Discussion and Conclusions

Our overlap‐kernel EPI takes inter‐shot overlap bands at arbitrary k‐space locations as another kind of ACS regions, for novel shot‐to‐shot phase extraction in one‐step. Particularly, this redefines self‐navigation—which traditionally required oversampling or at least recoverable k‐space center in every shot—and opens the door to new strategies in designing multi‐shot k‐space trajectories. Essentially, our method transfers elements in explicit kernel/map extraction techniques in PI (GRAPPA/ESPIRiT) to multi‐shot EPI, analogous to how low‐rank matrix completion approaches evolved from SAKE [38]/LOARKS [39, 58] into MUSSEL [24], and thus, surprisingly making PI and multi‐shot MRI much more thoroughly connected than before. Nevertheless, some conventional GRAPPA's characteristics [91] may not be directly suitable for our scope, since signal quality of the shot‐to‐shot calibration regions can be much worse than the fully‐sampled, non‐diffusion‐weighted, central k‐space ACS in PI.

Rather than advocating a specific sequence, we provide a general mechanism to “interlock” neighboring k‐space bands acquired in separate shots. In this paper, this principle successfully enables unconventional self‐navigated sequences (two‐shot and four‐shot mosaic) otherwise hindered by additional shot‐dependent eddy currents, upgrades the commonly used readout‐segmented and phase‐interleaved EPI in certain conditions, improves the use of 2D navigator data, and may provide other minor applications (e.g., extracting *B*_0_ maps given low‐to‐moderate *B*_0_ inhomogeneity). Note, phase‐interleaved EPI with kernel‐extracted 2D navigator corrections is similar to realigned GRAPPA [65, 66], but our implementation independently extracts phase kernels, which are loaded into a SENSE/ESPIRiT forward model reconstruction instead of unified GRAPPA interpolations.

The unconventional two‐shot/four‐shot center‐out mosaic EPI combines similar short‐TE benefits of spiral sampling [93] and low‐trajectory errors in Cartesian sampling, captures both diffusion‐ and hardware‐errors in high‐SNR self‐navigated ACS over refocused navigators, and appears suitable for multi‐shot diffusion scans when maximizing SNR is the priority (e.g., for high‐resolution and high *b*‐values applications). The point spread function due to T_2_ decay in these center‐out mosaic EPI is approximately symmetric about k‐space center, yielding apodization characteristics that differ from standard Cartesian EPI. In this case, low‐frequency image pixels that usually occupy most of the image support are acquired with minimized T_2_ decay, which potentially contributes to visibly higher SNR in reality. In the SNR‐limited regimes of our experiments, center‐out mosaic EPI produced remarkably clearer images once distortion was adequately corrected, facilitated by our shot‐to‐shot phase calibrations. Nevertheless, trade‐offs do still exist. The mosaic segmentation along phase‐encoding dimension leads to non‐minimized ESP, and may incur occasional blurring due to residue trajectory and *B*_0_ inhomogeneity errors (Figure S10) similar to spiral scans. However, while these limitations can still be reduced by high‐performance readout gradients [86] or optimized image reconstruction [94], irreversible SNR loss is never correctable. If minimization of ESP is prioritized, a two‐shot readout‐segmented EPI (center‐out along the readout direction) may also be employed with reasonable SNR in the self‐navigated ACS near the k‐space central region (along readout).

More commonly used multi‐shot diffusion sequences [37, 42] can also benefit. Given sufficient SNR, the self‐navigated readout‐segmented EPI (0.7 mm^2^) can reach identical or better (i.e., due to additional shot‐dependent eddy current calibration) reconstruction quality than 2D navigator correction (i.e., RESOLVE), with *b*‐values up to 1500 s/mm^2^ in 3T Prisma, and 2000 s/mm^2^ in 3T Cima.X scanners. The diffusion‐weighting limits might be further improved with fully utilized high‐performance gradients (e.g., shorter TE) [92] or neural network‐based kernel extraction [95]. In self‐navigated phase‐interleaved EPI, kernel‐based phase calibration in a central k‐space region [23, 71] always benefits from much higher SNR than in periphery of k‐space [24]. Along this path, the overlap‐kernel EPI, implemented as MUSE with kernel extraction (one‐step GRAPPA/ESPIRiT‐type operation applied to intermediate PI reconstruction), further unifies the two steps in JULEP [71] elegantly (structured low‐rank constraints and explicit phase mapping), and efficiently removes residue phase estimation errors in conventional MUSE. Nevertheless, in very strong diffusion‐weighting (> 3000 s/mm^2^) or ultrahigh‐field, both these conventional multi‐shot trajectories may suffer from critical SNR drop and increased SAR, for self‐navigation or 2D navigators (acquired after refocusing pulses) corrections.

In our implementations, the small overlap regions do not incur substantially increased sampling time, while the refocused navigators typically cost extra time of a few tens of milliseconds (e.g., comparing protocols in Figures S2 and S9). Empirically, a 7 × 7 kernel size appears sufficient in most of our experiments, while a larger kernel size may be considered for high *b*‐values (e.g., 3000 s/mm^2^, Figure S7), as reserved for future study. Regarding estimation accuracy, our kernel subspace analysis (i.e., singular‐value distribution) across k‐space overlap locations, diffusion‐weighted *b*‐values, and kernel sizes/thresholds confirm limitations of kernel extraction techniques originated from low‐SNR calibration regions, mainly due a combination of strong diffusion‐weighting, and peripheral k‐space locations [12]. This is problematic for outer k‐space bands in strong diffusion‐weighted readout‐segmented EPI, and might pose even stronger challenges for the most outer k‐space region in MUSSEL [24]. Therefore, the two‐shot/four‐shot mosaic EPI with short‐TE‐overlap regions at/close to the k‐space origin deserves more future investigations for high‐resolution, strong diffusion‐weighting scans using high‐performance gradient scanners.

In this work, only nominal trajectory re‐gridding followed by 1D‐FID‐based N/2 ghosts correction has been tested. Our inter‐shot phase calibration only removes relative image‐space phase variations or small k‐space displacements between shots. Consequently, strong and continuous eddy currents within each shot, if present, may disrupt the kernel interpolation modeling, resulting in shot‐to‐shot estimation errors. Theoretically, combining more realistic within‐shot trajectory corrections based on e.g., even‐odd GRAPPA kernels [64], continuous B_0_ kernels [59], or field camera [87] can improve quality in inter‐shot ACS regions and the resultant estimations. Additionally, the shot‐dependent amplitude fluctuation maps are also obtained but discarded for reconstruction, which, however, might become useful in future studies.

For higher sampling efficiency, our demonstrated sequences can be easily extended to SMS [96], or adapted for 3D volumetric scans with tailored overlap regions, to robustly remove primary [5, 6] or residue [90] (e.g., given motion compensation) shot‐to‐shot signal inconsistencies in different scenarios, well‐suited for high‐resolution diffusion‐weighted [97] or functional [6, 98, 99, 100] MRI scans. However, the most optimized multi‐shot EPI sequence may strongly depend on applications, involving considerations about achieved resolution, shot number, SNR, tolerable artifact patterns, acoustic prohibited bands, nerve stimulations, field strengths, gradient types, and product availability from the vendor. Generally, any sequences where different shots sample a small common k‐space region with sufficient SNR can benefit from this strategy to stabilize inter‐shot signals. Similar to PI, the robustness of self‐calibration in the form of kernels originates from an elegant property of the Fourier domain: smooth spatial modulation manifests as condensed k‐space kernels with a large number of shifts (i.e., averaging and stability). In MRI, these small and computationally efficient kernels can widely represent effects due to *B*_0_ [59, 60, 64, 101, 102] and *B*_1_ [55, 62, 95] magnetic fields, although they may encounter difficulties in cases of unusually abrupt spatial modulations (Figure S5). As such, we believe the proposed kernel extraction techniques [68] encapsulated by the generalized k‐space signal interpolations framework [60, 67] or ESPIRiT [56] theory will continue to find more applications [59] in future MRI developments.

## Supporting information


**Data S1:** mrm70196‐sup‐0001‐DataS1.pdf.
**Figure S1:** Similarity of calibration matrix formulation for auto‐calibration in parallel imaging and multi‐shot rs‐EPI. (A) Low‐resolution GRE scans used for calibrating parallel imaging. (B) A sliding mathematical window (green) shifts within the fully‐sampled central k‐space region, acquired by distinct RF receiver channels (represented by different colors), with each patch or neighborhood reshaped into a row in the calibration matrix. The diffusion fluctuation kernels or cardinal functions remain shift‐invariant as the sliding window shifts across different k‐space locations and RF receiver channels. (C) k‐space sampling trajectory in rs‐EPI where two readout segments in consecutive shots overlapped by approximately, for example, 10–20 pixels. The readout gradient polarity is flipped between overlapped segments to ensure consistent T_2_ or T_2_* signal decay at the overlapped k‐space locations. (D) In the rs‐EPI case, a sliding window (green) shifts within the overlapping region between two segments sampled separately in consecutive shots across all RF receivers, and each neighborhood in a shift is reshaped into a row in the calibration matrix. In this rs‐EPI sequence, only two readout segments partially overlap, so each row in the calibration matrix contains two reshaped patches. Data from different k‐space locations and RF receiver channels together form a “shift‐invariant” dimension for the cardinal functions. (E) A generalized calibration matrix highlights the mathematical similarity between estimating RF receiver sensitivity maps and shot‐dependent phase fluctuation maps. The shifts of the window create a “shift‐invariant” dimension (the vertical dimension of the matrix), which improves the conditioning of the calibration matrix and makes kernel estimation more robust.
**Figure S2:** A full‐version of Figure 4 in manuscript. Additionally, the difference maps between self‐navigation and 2D navigator corrected reconstruction (normalized by the latter) are shown in percentage. Note, neither reconstruction is the ground‐truth, so these difference maps only reflect their reconstruction variations in fine structures. Moreover, the kernel subspaces for self‐navigated ACS and separately acquired 2D navigator ACS are shown. The singular‐value distributions are normalized with maximum value of 1, with the self‐navigation on the left (blue), the 2D navigator on the right (black), and the subspace thresholding indicated by the red dash line. In two‐shot and four‐shot mosaic EPI, kernel subspaces for self‐navigation are more low‐rank than the 2D navigators, indicating higher SNR in self‐navigated ACS due to shorter TE. In phase‐interleaved EPI, self‐navigated ACS also exhibit a more low‐rank property than 2D navigator, possibly because the intermediate PI reconstruction at central k‐space (50 × 50) has shorter TE than navigator echoes after refocused pulses. However, this does not always translate into improved estimation accuracy, as low‐rank distribution in kernel subspace is a too general assumption, and might not specifically capture all errors in self‐navigated ACS, for example, resulted from failed intermediate PI reconstructions for individual shots (e.g., residue ghosts, in reconstruction of 8× undersampled k‐space).
**Figure S3:** Acoustic resonance distributions and prohibited bands (rectangles) for 3T Siemens Prisma^fit^, which shows the readout‐segmented EPI (Figure 4 in manuscript) with shortened ESP (by removing k‐space overlaps) was hindered by acoustic resonance constraints, not necessary the gradient power. (A) Each shot includes 16 additional overlapped readout points for self‐navigation. (B) The k‐space shot‐to‐shot overlaps were removed, and the saved sampling time was converted to a reduction of 0.08 ms in ESP (unfortunately, hits the prohibited bands), while maintaining similar readout gradient performance. In practice, the prolonged ESP in rs‐EPI may not result from additional k‐space overlaps, but scanner‐specific prohibited acoustic bands. The 0.54 ms ESP in (A) can be theoretically shortened by increasing readout gradient amplitude, but is finally prohibited due to potential mechanical damage of gradients. In this protocol, the additional 16 overlapped readout points do not cause extra scan time, but just slightly raises the gradient performance, well below the hardware (80mT/m) and PNS limit. This sequence limitation is common for rs‐EPI, but may be mitigated by applying oscillating phase‐encoding gradients during low‐bandwidth readout as in Wave‐EPI.
**Figure S4:** The kernel extraction approach can also benefit phase extraction from overlapped data at k‐space center. Here, the eigenvalue approach (ESPIRiT‐type operation) was used with 7 × 7 kernel size. (A) Shot‐to‐shot phase maps can be obtained via kernel extraction from 2D navigators, which was more robust than naive smoothing and zero‐filled to high‐resolution grid. Sum images with three diffusion directions are shown. This is similar to realigned GRAPPA, but with phase kernels independently extracted and loaded into a SENSE/ESPIRiT forward model, instead of unified GRAPPA interpolations. (B) Comparing different approaches to extract phase maps from intermediate reconstruction of each phase‐interleaved shot. Sum images consisting of 12 diffusion directions are shown. Occasionally, errors in the original MUSE (total variation smoothing of image‐space phase) can be observed, which are eliminated by our approach—MUSE with kernel extraction. This is similar to improved extrapolation of RF sensitivity maps by shifted kernels in ESPIRiT, over an image‐space operation.
**Figure S5:** Four‐shot mosaic EPI sequence (*B*_0_ inhomogeneity sensitive) with different approaches to extract *B*_0_ map from the central region of k‐space of the GRE data with different TE. The sequence has a moderate ESP 0.7 ms (effective 0.23 ms), but its trajectory remains sensitive to *B*_0_ offset similar to spiral‐out trajectory. Compared to naive smoothing with Kaiser filter in k‐space, the kernel extraction approach with large kernel size (20 × 20) appears robust to remove noise around the skull. This worked for low‐to‐moderate *B*_0_ inhomogeneity at 3T in our tests, but may be difficult to capture very abrupt image‐space *B*_0_ variations (e.g., 7T or above) with too large kernels (i.e., computational expensive). The computational time for large (e.g., 20 × 20) kernel can be substantially increased compared to a usual size (7 × 7), and also depends on the calibration region size and the finally extrapolated matrix size (if using ESPIRiT‐type operation).
**Figure S6:** Reconstruction quality with respect to kernel size, subspace threshold, and low‐resolution apodization. (A) impact of kernel size and subspace threshold. (B) Effects of apodization on each EPI segment and the corresponding inverse filter. The ex vivo phantom had no physiological phase fluctuations, and can only suffer from shot‐dependent system imperfections. The diffusion‐weighted images are shown as the sum image for a total of 20 diffusion directions.
**Figure S7:** Diffusion phase fluctuations represented as image‐space maps and k‐space kernels, for *b*‐values 1000, 2000, and 3000 s/mm^2^. Five repetitions (Shot #1–5) of single‐shot diffusion‐weighted EPI were performed for different *b*‐values. Row 1: The 2D image phase maps for Shot#1. Row 2: The Fourier transform of image‐space phase maps, as the diffusion phase kernels (absolute values displayed), for Shot#1. A commonly used 7 × 7 kernel window (red rectangle) illustrates the portion of diffusion phase information captured by this kernel size across diffusion weighting. Row 3–4: Two 1D distribution of the k‐space kernels are plotted to indicate the k‐space range of diffusion phases, with the kernel size marked at half of the maximum values. As *b*‐value increases, the k‐space diffusion kernels become more spread. Note that, due to sharp image‐space truncation from background (finite object support), the k‐space kernel distributions might appear more spread than they actually are.
**Figure S8:** Readout‐segmented EPI sequences with increasing diffusion‐weighting, to visualize the SNR dependency on *b*‐values ranging from 0 to 1500 s/mm^2^. The diffusion‐weighted images are shown as the sum images for a total of 20 diffusion directions.
**Figure S9:** The same diffusion‐weighted dataset (1000 s/mm^2^, 32 diffusion directions, and 2 averages) in manuscript's Figure 9, with more slice positions, and uncorrected multi‐shot reconstructions to reveal possible artifact patterns which may be helpful for protocol selections. The ESPs are the same as the corresponding sequences with identical trajectories in manuscript's Figure 5.
**Figure S10:** The same diffusion‐weighted dataset in manuscript's Figure 5, with also non‐diffusion‐weighted images shown, in another slice position. The self‐navigated sum images consisting of three diffusion directions, and coefficient‐of‐variation maps for one diffusion direction over 10 repetitions are shown. In this slice with potentially stronger *B*_0_ inhomogeneity, the four‐shot mosaic self‐navigated EPI appears blurred, although its CV maps remain smaller, relative to five‐shot readout‐segmented and phase‐interleaved EPI. Similar to other slice positions, additional artifacts or residue ghosts can be found in the two‐shot mosaic and five‐shot phase‐interleaved EPI with 2D navigator corrections, as well as the conventional MUSE. These are well‐minimized in our overlap‐kernel self‐navigation techniques. Note, the effective ESP (not ESP) are shown, as the ESP divided by k‐space undersampling (including possible phase‐interleaved) factor in each shot.
**Figure S11:** The same diffusion‐weighted dataset in manuscript's Figure 5, with also non‐diffusion‐weighted images shown, in another slice position. The self‐navigated sum images consisting of 3 diffusion directions, and coefficient‐of‐variation maps for one diffusion direction over 10 repetitions are shown. Similar to other slice positions, additional artifacts or residue ghosts can be found in the two‐shot mosaic and five‐shot phase‐interleaved EPI with 2D navigator corrections, as well as the conventional MUSE. These are well‐minimized in our overlap‐kernel self‐navigation techniques. Note, the effective ESP (not ESP) are shown, as the ESP divided by k‐space undersampling (including possible phase‐interleaved) factor in each shot.
**Figure S12:** The same diffusion‐weighted dataset in manuscript's Figure 5, with also non‐diffusion‐weighted images shown, in another slice position. The self‐navigated sum images consisting of 3 diffusion directions, and coefficient‐of‐variation maps for one diffusion direction over 10 repetitions are shown. In this slice with potentially stronger *B*_0_ inhomogeneity, the four‐shot mosaic self‐navigated EPI appears blurred, although its CV maps remain smaller, relative to five‐shot readout‐segmented and phase‐interleaved EPI. Similar to other slice positions, additional artifacts or residue ghosts can be found in the two‐shot mosaic and five‐shot phase‐interleaved EPI with 2D navigator corrections, as well as the conventional MUSE. These are well‐minimized in our overlap‐kernel self‐navigation techniques. Note, the effective ESP (not ESP) are shown, as the ESP divided by k‐space undersampling (including possible phase‐interleaved) factor in each shot.
**Figure S13:** The same diffusion‐weighted dataset in manuscript's Figure 5, with also non‐diffusion‐weighted images shown, in another slice position. The self‐navigated sum images consisting of 3 diffusion directions, and coefficient‐of‐variation maps for one diffusion direction over 10 repetitions are shown. In this slice with potentially stronger *B*_0_ inhomogeneity, the four‐shot mosaic self‐navigated EPI appears blurred, although its CV maps remain smaller, relative to five‐shot readout‐segmented and phase‐interleaved EPI. Similar to other slice positions, additional artifacts or residue ghosts can be found in the two‐shot mosaic and five‐shot phase‐interleaved EPI with 2D navigator corrections, as well as the conventional MUSE. These are well‐minimized in our overlap‐kernel self‐navigation techniques. Note, the effective ESP (not ESP) are shown, as the ESP divided by k‐space undersampling (including possible phase‐interleaved) factor in each shot.
**Table S1:** SE‐EPI in Figures 4 and S2.
**Table S2:** SE‐EPI in Figures 5 and S10–S13.
**Table S3:** Readout‐segmented EPI in Figures 6 and 7.
**Table S4:** SE‐EPI with long and short diffusion‐weighted gradients in a 3T Siemens Cima.X scanner, as in Figure 8.
**Table S5:** Self‐navigated SE‐EPI in Figures 9 and S9.
**Table S6:** Self‐navigated GE‐EPI in Figure 9.
**Table S7:** EPI in Figure S4.
**Table S8:** GRE in Figure S5.
**Table S9:** EPI in Figure S5.
**Table S10:** SE‐EPI for tuning kernel, in Figure S6A.
**Table S11:** SE‐EPI for evaluating filter and inverse filter, in Figure S6B.
**Table S12:** SE‐EPI in Figure S7.
**Table S13:** SE‐EPI for evaluating reconstruction with increasing diffusion‐weighting, in Figure S8.

## Data Availability

The example codes and data are available on Zenodo (https://zenodo.org/records/17704660). The example codes are also available on Github (https://github.com/ruitianwater/OverlapKernelEPI_v1.0) and the HarmonizedMRI website (https://harmonizedmri.github.io/projects/). Simplified codes would also be in the demo folder of Pulseq (https://pulseq.github.io/).
