# Supplementary material for: Overlap‐Kernel EPI: Estimating MRI Shot‐to‐Shot Phase Variations by Shifted‐Kernel Extraction From Overlap Regions at Arbitrary k‐Space Locations
Source: Magn Reson Med. 2025 Dec 15;95(4):2131–52. doi: 10.1002/mrm.70196 (PMC12850564; doi:10.1002/mrm.70196)
Supplement: Supplementary file 1 — Data S1: mrm70196‐sup‐0001‐DataS1.pdf. Figure S1: Similarity of calibration matrix formulation for auto‐calibration in parallel imaging and multi‐shot rs‐EPI. (A) Low‐resolution GRE scans used for calibrating parallel imaging. (B) A sliding mathematical window (green) shifts within the fully‐sampled central k‐space region, acquired by distinct RF receiver channels (represented by different colors), with each patch or neighborhood reshaped into a row in the calibration matrix. The diffusion fluctuation kernels or cardinal functions remain shift‐invariant as the sliding window shifts across different k‐space locations and RF receiver channels. (C) k‐space sampling trajectory in rs‐EPI where two readout segments in consecutive shots overlapped by approximately, for example, 10–20 pixels. The readout gradient polarity is flipped between overlapped segments to ensure consistent T2 or T2* signal decay at the overlapped k‐space locations. (D) In the rs‐EPI case, a sliding window (green) shifts within the overlapping region between two segments sampled separately in consecutive shots across all RF receivers, and each neighborhood in a shift is reshaped into a row in the calibration matrix. In this rs‐EPI sequence, only two readout segments partially overlap, so each row in the calibration matrix contains two reshaped patches. Data from different k‐space locations and RF receiver channels together form a “shift‐invariant” dimension for the cardinal functions. (E) A generalized calibration matrix highlights the mathematical similarity between estimating RF receiver sensitivity maps and shot‐dependent phase fluctuation maps. The shifts of the window create a “shift‐invariant” dimension (the vertical dimension of the matrix), which improves the conditioning of the calibration matrix and makes kernel estimation more robust. Figure S2: A full‐version of Figure 4 in manuscript. Additionally, the difference maps between self‐navigation and 2D navigator corrected reconstruction (nor [file MRM-95-2131-s001.pdf]

Supporting Information for

Overlap-Kernel EPI: Estimating MRI Shot-to-Shot Phase Variations by Shifted-Kernel Extraction From Overlap Regions at Arbitrary k-Space Locations

Rui Tian[1]*, Martin Uecker[2,3], Maxim Zaitsev[4], Klaus Scheffler[1,5]

[1]High-Field MR center, Max Planck Institute for Biological Cybernetics, Tübingen, Germany [2]Institute of Biomedical Imaging, Graz University of Technology, Graz, Austria [3]BioTechMed-Graz, Graz, Austria [4]Division of Medical Physics, Department of Diagnostic and Interventional Radiology, University Medical Center Freiburg, Faculty of Medicine, University of Freiburg, Freiburg, Germany [5]Department for Biomedical Magnetic Resonance, University of Tübingen, Tübingen, Germany

*corresponding author: rui.tian@tuebingen.mpg.

This Supporting Information documents additional technical information about our overlap-kernel approaches (i.e., kernel extraction algorithms) used for extracting shot-to-shot phase variations in MRI.

1 ACS regions in parallel imaging and multi-shot EPI

Figure S1 illustrates the similarity and difference in defining ACS regions and formulating calibration matrix in parallel imaging and multi-shot EPI.

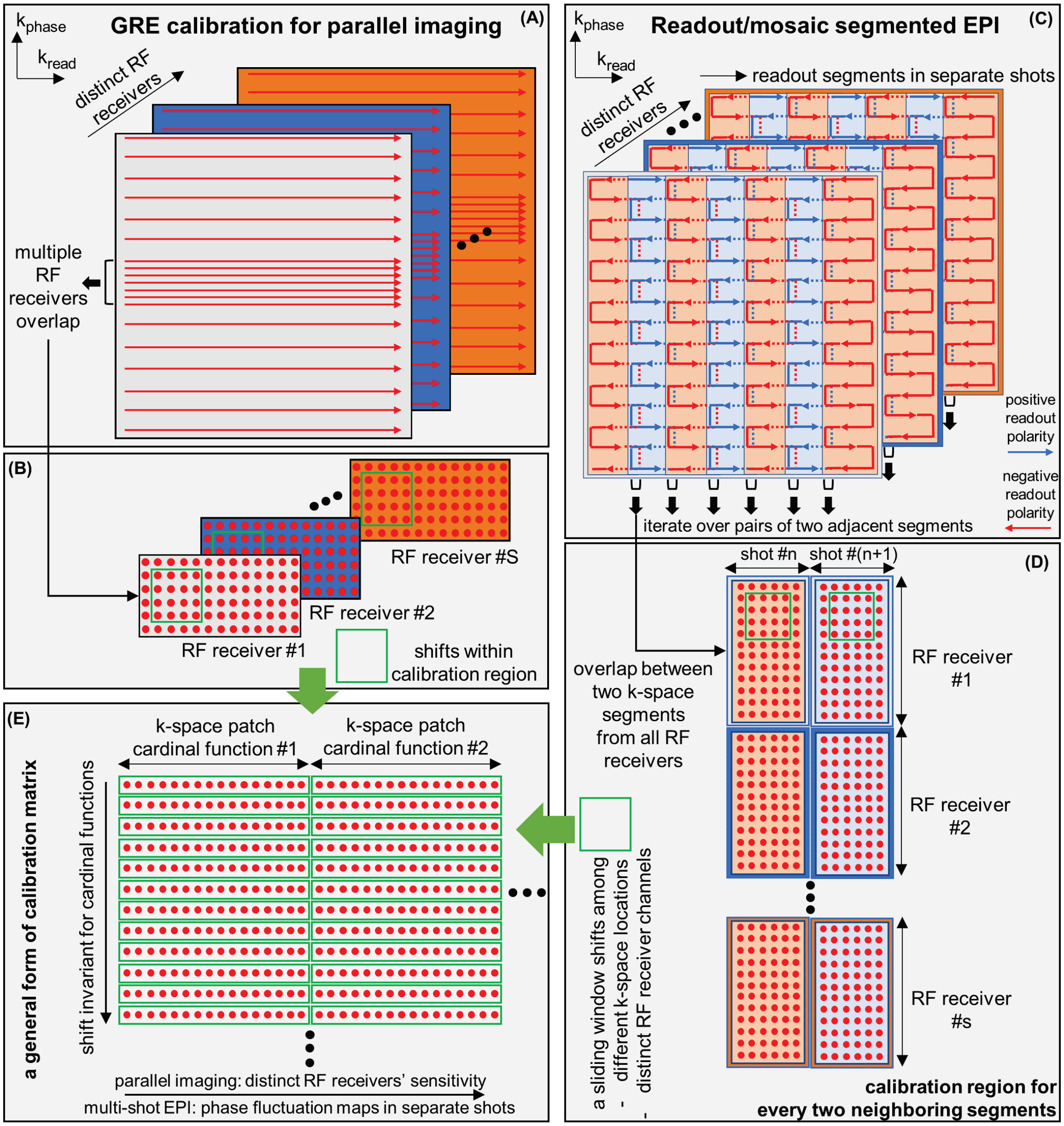

Figure S1. Similarity of calibration matrix formulation for auto-calibration in parallel imaging and multi-shot rs-EPI. (A) Low-resolution GRE scans used for calibrating parallel imaging. (B) A sliding mathematical window (green) shifts within the fully-sampled central k-space region, acquired by distinct RF receiver channels (represented by different colors), with each patch or neighborhood reshaped into a row in the calibration matrix. The diffusion fluctuation kernels or cardinal functions remain shift-invariant as the sliding window shifts across different k-space locations and RF receiver channels. (C) k-space sampling trajectory in rs-EPI where two readout segments in consecutive shots overlapped by approximately e.g. 10-20 pixels. The readout gradient polarity is flipped between overlapped segments to ensure consistent T_2 or T_2* signal decay at the overlapped k-space locations. (D) In the rs-EPI case, a sliding window (green) shifts within the overlapping region between two segments sampled separately in consecutive shots across all RF receivers, and each neighborhood in a shift is reshaped into a row in the calibration matrix. In this rs-EPI sequence, only two readout segments partially overlap, so each row in the calibration matrix contains two reshaped patches. Data from different k-space locations and RF receiver channels together form a "shift-invariant" dimension for the cardinal functions. (E) A generalized calibration matrix highlights the mathematical similarity between estimating RF receiver sensitivity maps and shot-dependent phase fluctuation maps. The shifts of the window create a "shift-invariant" dimension (the vertical dimension of the matrix), which improves the conditioning of the calibration matrix and makes kernel estimation more robust.

2 Robust and versatile self-navigation for different trajectories

Figure S2 provides a full-version of Figure 4 in the manuscript, with additional difference maps between self-navigated and 2D navigator corrected reconstructions (normalized by 2D navigator corrections), and their subspace thresholds.

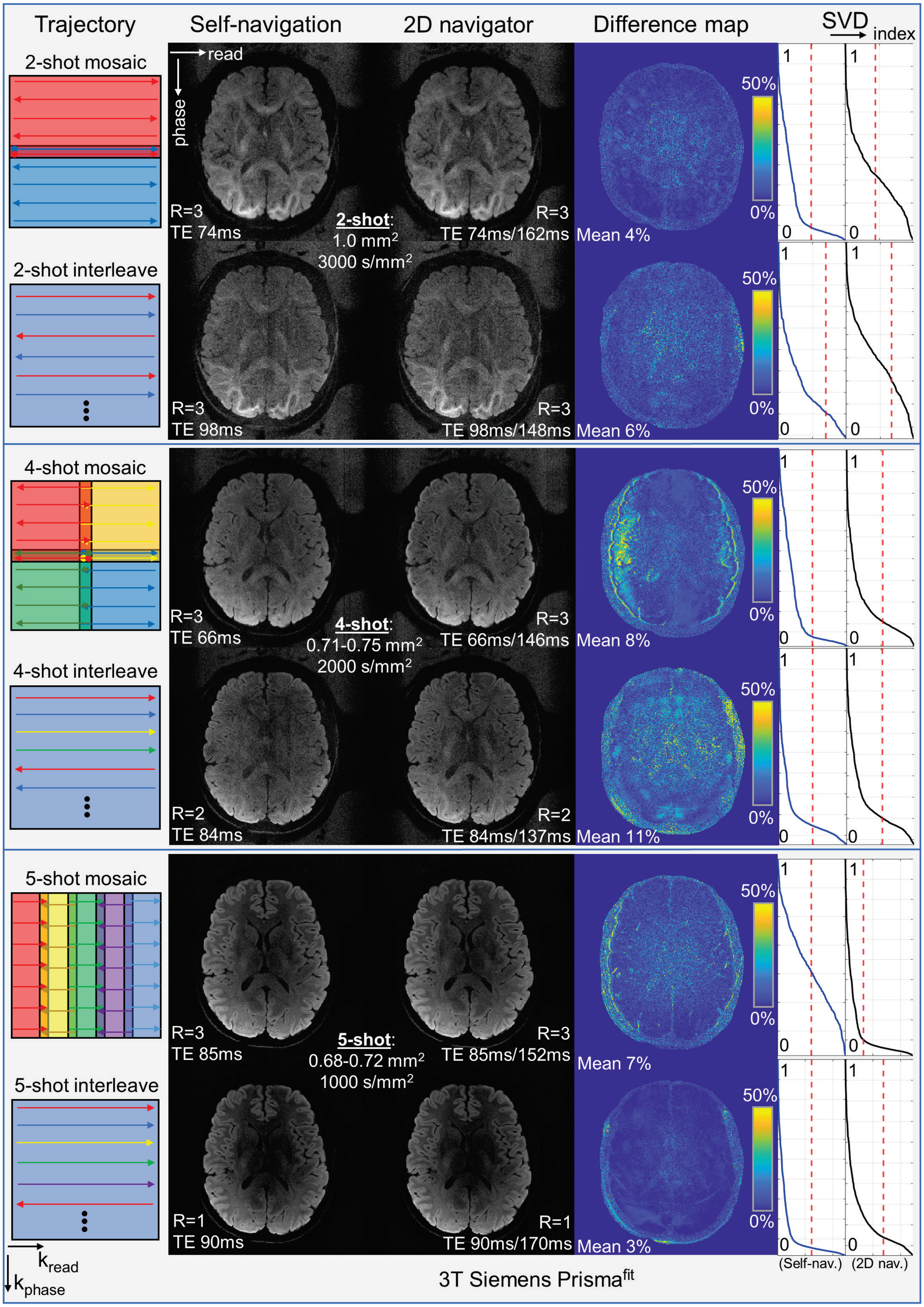
Trajectory
Self-navigation
2D navigator
Difference map
SVD
index
2-shot mosaic
read
phase
R=3
TE 74ms
2-shot:
1.0 mm²
3000 s/mm²
R=3
TE 74ms/162ms
Mean 4%
50%
0%
2-shot interleave
R=3
TE 98ms
R=3
TE 98ms/148ms
Mean 6%
4-shot mosaic
R=3
TE 66ms
4-shot:
0.71-0.75 mm²
2000 s/mm²
R=3
TE 66ms/146ms
Mean 8%
4-shot interleave
R=2
TE 84ms
R=2
TE 84ms/137ms
Mean 11%
5-shot mosaic
R=3
TE 85ms
5-shot:
0.68-0.72 mm²
1000 s/mm²
R=3
TE 85ms/152ms
Mean 7%
5-shot interleave
R=1
TE 90ms
R=1
TE 90ms/170ms
Mean 3%
k_read
k_phase
3T Siemens Prisma^fit
(Self-nav.)
(2D nav.)

Figure S2. A full-version of Figure 4 in manuscript. Additionally, the difference maps between self-navigation and 2D navigator corrected reconstruction (normalized by the latter) are shown in percentage. Note, neither reconstruction is the ground-truth, so these difference maps only reflect their reconstruction variations in fine structures. Moreover, the kernel subspaces for self-navigated ACS and separately-acquired 2D navigator ACS are shown. The singular-value distributions are normalized with maximum value of 1, with the self-navigation on the left (blue), the 2D navigator on the right (black), and the subspace thresholding indicated by the red dash line. In 2-shot and 4-shot mosaic EPI, kernel subspaces for self-navigation are more low-rank than the 2D navigators, indicating higher SNR in self-navigated ACS due to shorter TE. In phase-interleaved EPI, self-navigated ACS also exhibit a more low-rank property than 2D navigator, possibly because the intermediate PI reconstruction at central k-space (50x50) has shorter TE than navigator echoes after refocused pulses. However, this does not always translate into improved estimation accuracy, as low-rank distribution in kernel subspace is a too general assumption, and might not specifically capture all errors in self-navigated ACS, e.g., resulted from failed intermediate PI reconstructions for individual shots (e.g., residue ghosts, in reconstruction of 8x undersampled k-space).

Figure S3 provides evidence for the readout-segmented EPI in Figure S2 and Figure 4, that the ESP or effective ESP cannot be further shortened due to acoustic resonance prohibited bands, rather than gradient performances. For future work, this sequence limitation of rs-EPI may be mitigated by applying oscillating phase-encoding gradients during its low-bandwidth readout, as in Wave-EPI.

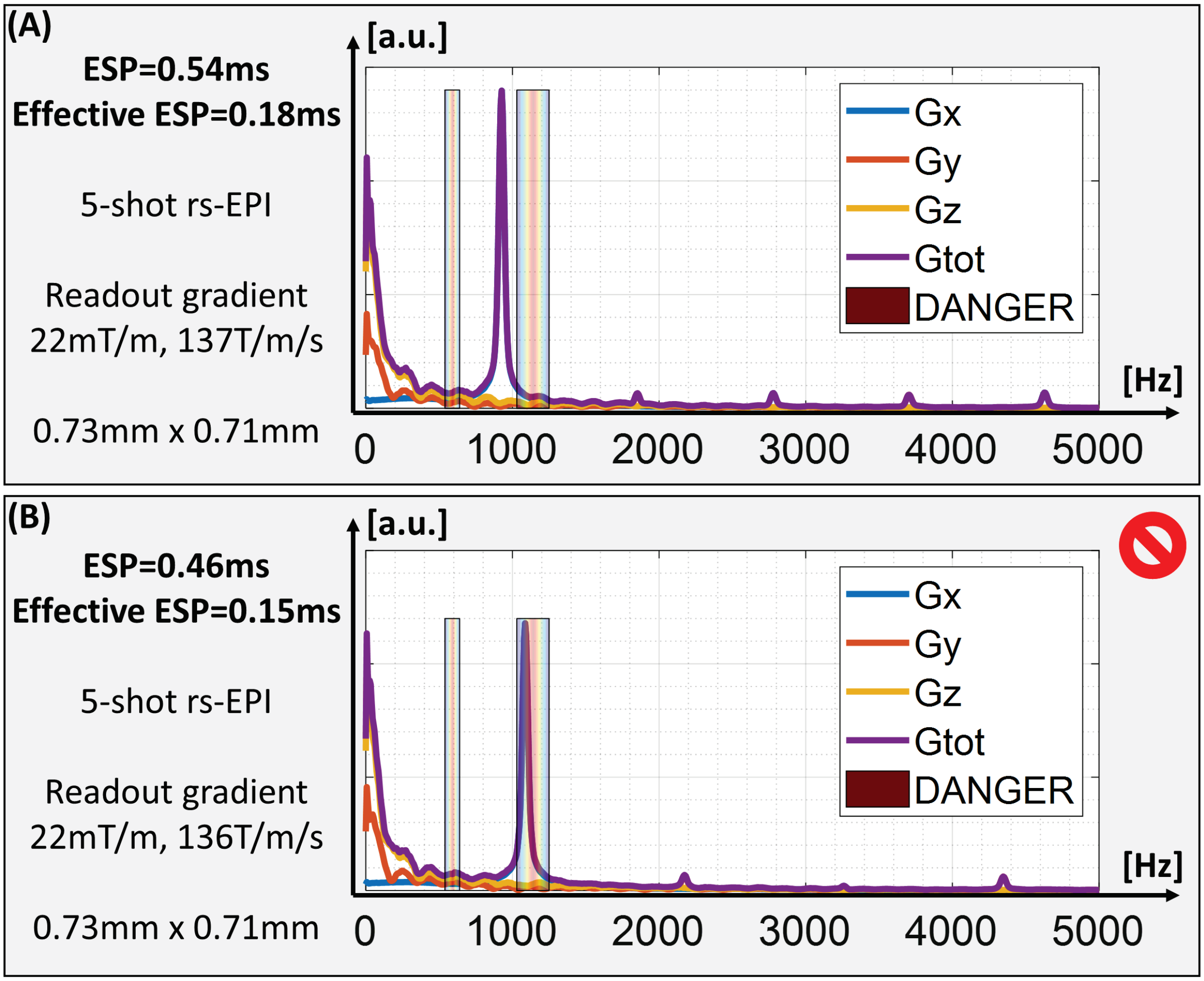

Figure S3. Acoustic resonance distributions and prohibited bands (rectangles) for 3T Siemens Prisma^{fit}, which shows the readout-segmented EPI (Figure 4 in manuscript) with shortened ESP (by removing k-space overlaps) was hindered by acoustic resonance constraints, not necessary the gradient power. (A) Each shot includes 16 additional overlapped readout points for self-navigation. (B) The k-space shot-to-shot overlaps were removed, and the saved sampling time was converted to a reduction of 0.08 ms in ESP (unfortunately, hits the prohibited bands), while maintaining similar readout gradient performance. In practice, the prolonged ESP in rs-EPI may not result from additional k-space overlaps, but scanner-specific prohibited acoustic bands. The 0.54ms ESP in (A) can be theoretically shortened by increasing readout gradient amplitude, but is finally prohibited due to potential mechanical damage of gradients. In this protocol, the additional 16 overlapped readout points do not cause extra scan time, but just slightly raises the gradient performance, well below the hardware (80mT/m) and PNS limit. This sequence limitation is common for rs-EPI, but may be mitigated by applying oscillating phase-encoding gradients during low-bandwidth readout as in Wave-EPI.

3 Kernel extraction from overlapped k-space central region

As special cases, we can also apply our overlap-kernel approaches to overlapped data across different shots centered at the k-space origin, similar to GRAPPA/ESPIRiT applied to the central ACS regions across multiple RF receiver channels in parallel imaging.

As seen in the upper part of Figure S4, for a 2-shot mosaic EPI with relatively long ESP (effective 0.36ms) and b-value 2000 s/mm^2, the 2D navigator k-space data overlapped (pixel-size 222x37) at the k-space center, which was sampled after imaging echoes with consistent ESP that may suffer from geometric distortion. The shot-to-shot phase fluctuation maps can be obtained by either naive apodization followed by zero-filling the k-space data, or k-space kernel extraction as implemented by the eigenvalue approach. The naive-apodization-and-zero-filling approach did not adequately remove navigator errors, largely caused by strong local distortion along the low-resolution phase-encoding direction, as highlighted in the zoomed image. In contrast, taking navigator data as ACS regions similar to realigned GRAPPA, the kernel extraction operation produced a smoother phase map, leading to improved reconstruction without the navigator's errors. The low-rank property of kernel subspace implied a high capacity for robustly extracting the phase maps, well-represented by only a few singular-vectors.

As seen in the bottom part of Figure S4, our kernel extraction techniques can improve a 6-shot self-navigated phase-interleaved EPI with short ESP (effective 0.18ms) and b-value 2500 s/mm^2, termed "MUSE with kernel". The shot-to-shot phase fluctuation maps were estimated from intermediate PI reconstruction for each shot of interleave. The final images were reconstructed using all shots of data and phase fluctuation maps, and the RF sensitivity maps. The reconstruction was compared with the one robustly extracting shot-dependent phase kernels from separate 2D navigator scans.

The image reconstructed with 2D navigator correction exhibited a resolution comparable to that of the other self-navigated reconstruction. The original MUSE reconstruction is shown, which estimated shot-dependent phase maps from intermediate parallel imaging reconstruction using an image-space total-variation (TV) operation. Although the achieved resolution was visibly similar to the reconstruction with 2D navigator correction, local blur was occasionally observed as shown in the zoomed-in region. In our

proposed overlap-kernel EPI implementation as “MUSE with kernel”, from the intermediate PI reconstructed k-space data, kernel extraction was applied (here, ESPIRiT-type operation) to a central 50x50 k-space region. Phase fluctuation maps were modeled as 7x7 kernels shifted over 1800 times. This presumably reduced estimation errors compared to image-space smoothing that only applies averaging over several neighboring pixels. Consequently, the local blur in the original MUSE reconstruction was effectively removed. This approach elegantly combines the two steps of “joint usage of structured low-rank constraints and explicit phase mapping” in JULEP into one-step convergence, explicitly extracting inter-shot phase maps, without the need for iterative low-rank matrix completion or further redundant image-space smoothing operations.

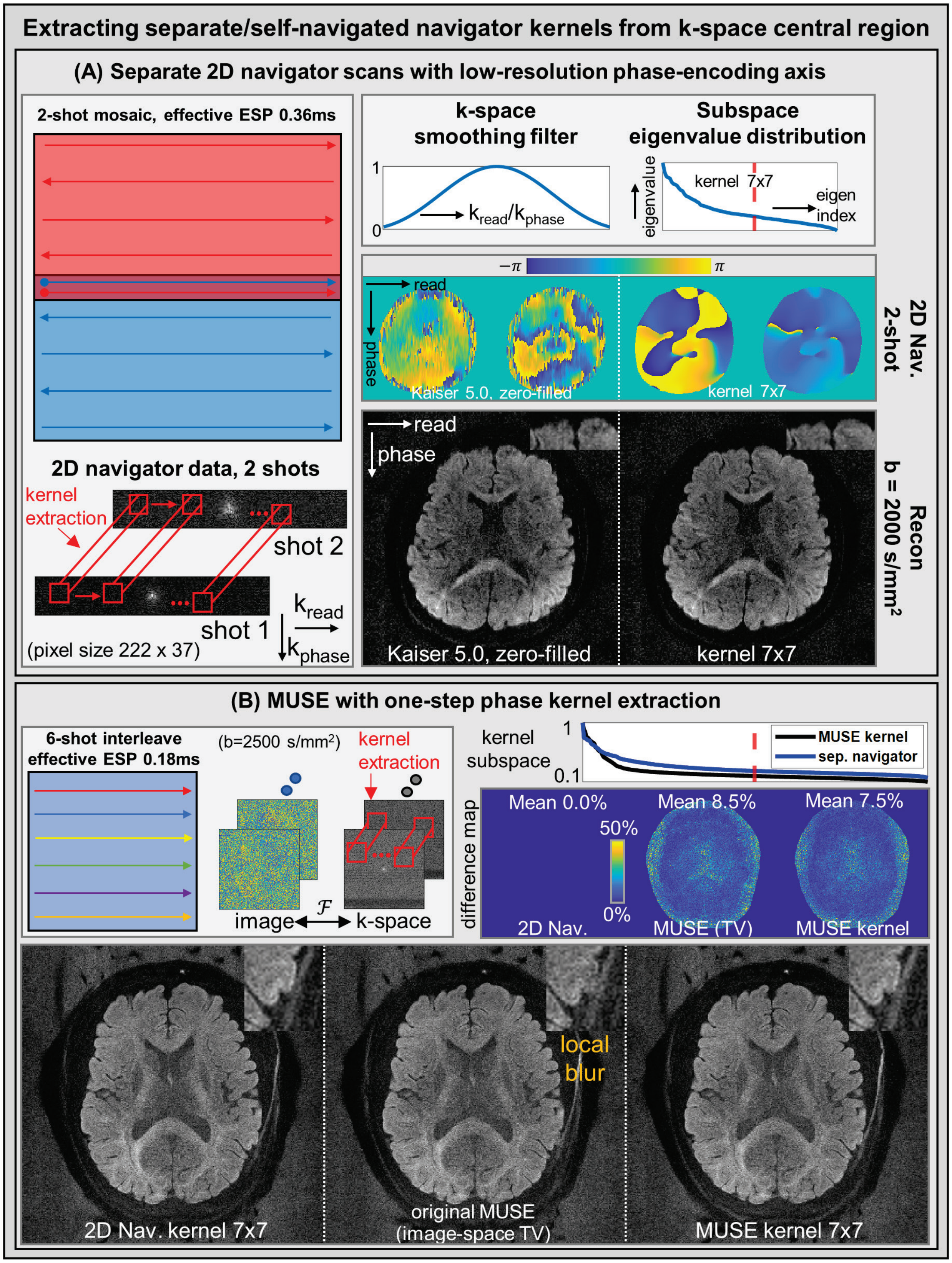

Figure S4. The kernel extraction approach can also benefit phase extraction from overlapped data at k-space center. Here, the eigenvalue approach (ESPIRiT-type operation) was used with 7x7 kernel size. (A) Shot-to-shot phase maps can be obtained via kernel extraction from 2D navigators, which was more robust than naive smoothing and zero-filled to high-resolution grid. Sum images with 3 diffusion directions are shown. This is similar

to realigned GRAPPA, but with phase kernels independently extracted and loaded into a SENSE/ESPIRiT forward model, instead of unified GRAPPA interpolations. (B) Comparing different approaches to extract phase maps from intermediate reconstruction of each phase-interleaved shot. Sum images consisting of 12 diffusion directions are shown. Occasionally, errors in the original MUSE (total variation smoothing of image-space phase) can be observed, which are eliminated by our approach – MUSE with kernel extraction. This is similar to improved extrapolation of RF sensitivity maps by shifted kernels in ESPIRiT, over an image-space operation.

4 Kernel extraction at k-space center for B_0 maps

As in Figure S5, our proposed kernel extraction approaches can robustly extract a smooth B_0 map from GRE data centered at the k-space origin with two distinct TE. In this case, the 4-shot mosaic EPI sequence was sensitive to B_0 field inhomogeneity, primarily due to the different phase encoding directions in each shot. This can be seen in the non-diffusion-weighted image.

Two 2D GRE scans with TE difference of 3ms were used to generate B_0 phase offset maps. These maps were incorporated into the forward model of PI reconstruction, aiming at distortion-free reconstruction. Three strategies are compared for producing a B_0 map, in which the kernel extraction was implemented with the eigenvalue approach.

First, a k-space filter (Kaiser 5.0) can be applied to smooth the GRE data. However, noise remains in the air between skull and brain (the inner noise ring), resulting in a noisy B_0 map at the brain edge.

Second, a 6x6 kernel can be extracted from the k-space central region of the GRE data, given a low-rank kernel subspace. In this way, the noise between skull and brain is effectively removed. However, some residue distortion persists.

Third, a 20x20 kernel can be extracted from the k-space central region of the GRE data. The nominal resolution of the extracted B_0 map is enhanced compared to the 6x6 kernel, while the low-rank proeperty of the kernel subspace is preserved. This not only removes noise but also captures stronger

image-space B_0 variations, and consequently, corrects the residue distortion observed in the smaller kernel extraction.

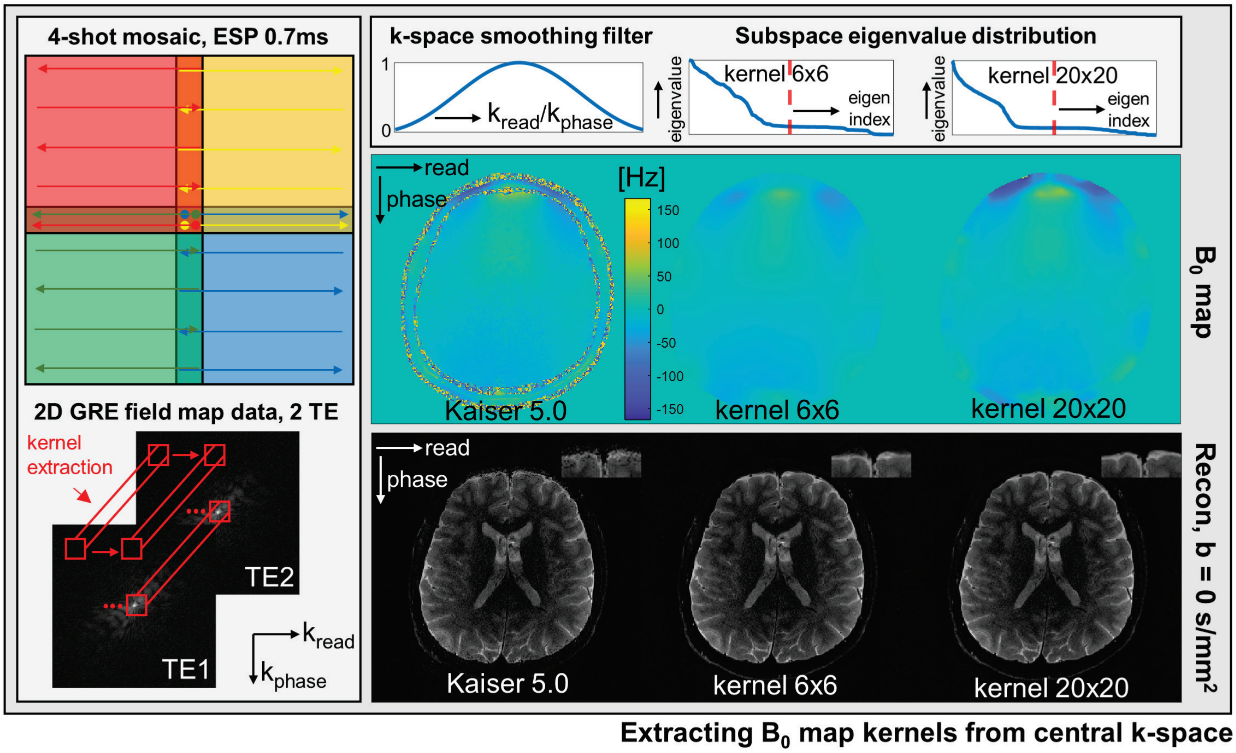

Figure S5. Four-shot mosaic EPI sequence (B_0 inhomogeneity sensitive) with different approaches to extract B_0 map from the central region of k-space of the GRE data with different TE. The sequence has a moderate ESP 0.7ms (effective 0.23ms), but its trajectory remains sensitive to B_0 offset similar to spiral-out trajectory. Compared to naive smoothing with Kaiser filter in k-space, the kernel extraction approach with large kernel size (20x20) appears robust to remove noise around the skull. This worked for low-to-moderate B_0 inhomogeneity at 3T in our tests, but may be difficult to capture very abrupt image-space B_0 variations (e.g., 7T or above) with too large kernels (i.e., computational expensive). The computational time for large (e.g., 20x20) kernel can be substantially increased compared to a usual size (7x7), and also depends on the calibration region size and the finally extrapolated matrix size (if using ESPIRiT-type operation).

5 Tuning kernel size, subspace threshold, filter and inverse filter.

Figure S6 demonstrates how subspace algorithm parameters affects the reconstruction quality of multi-shot scans, using the eigenvalue approach (i.e. ESPIRiT-type operation) as an example to estimate shot-

to-shot phase variations. We focus on parameters such as kernel size, subspace threshold and optional apodization of low-resolution readout segments. Similar subspace truncation can be applied to the approach of direct estimation of interpolation kernels (i.e. GRAPPA-type operation), for example, by using TSVD to invert the k-space interpolation relationships. However, the detailed differences in subspace thresholding between these two approaches are beyond the scope of this paper. For simplicity, we used the Moore-Penrose inverse (e.g. “pinv” in MATLAB) for the GRAPPA-type operation, as in Figure 7 of the manuscript.

5.1 Kernel size and subspace threshold

Figure S6A shows results from a 5-shot readout-segmented EPI acquisition (0.7mm^2) with large number (i.e. 27) of pixels overlapping along the readout dimension. Reconstructions were tested using small (i.e. 4x4) and large (i.e. 23x23) kernel sizes. In practice, large kernel size (e.g., 20) is not necessary for most diffusion-weighting scans (mostly-used, 7x7 kernel) in this paper, and only appears useful for very abrupt image-space variations, e.g., processing B_0 inhomogeneity maps as in Figure S5.

- Small kernel (4x4)
  Tuning subspace truncation thresholding has a strong effect. Truncating too many (90%, left column) or too few (5%, right column) singular vectors from SVD of the calibration matrix results in blurring (“blur” in the images), indicating poor estimation of the shot-dependent phase maps. A moderate truncation (65%, middle column) preserves image sharpness.
- Large kernel (23x23)
  Both 90% and 65% truncations yield quality similar to the case for 4x4 kernel with 65% truncation. However, minimal truncation again causes blurring. The larger kernel improves signal-noise subspace separability due to a sharper drop-off in the singular-value distribution, enhancing robustness to threshold variation.
- Diffusion-weighting
  Non-diffusion-weighted scans (i.e. b=0 s/mm^2, black, green) exhibits more concentrated singular-value distribution (i.e., low-rank), compared to diffusion-weighted scans (i.e., blue, magenta), reflecting higher SNR and more redundant encoding for kernel in non-diffusion-weighted scans.

5.2 Apodization before image-space phase map multiplication

Figure S6B evaluates the impact of filtering low-resolution readout segments before image-space phase fluctuations correction, compensated by the inverse filter in the final k-space. These apodization may not be needed in the second reconstruction approach in Section 2.4 of the manuscript, which, however, could take longer computational time to combine shots of data.

The upper row shows that, shot-dependent eddy currents can also be captured and removed by our proposed kernel extraction approaches, and may benefit from optional low-resolution apodizations.

Two 7-shot readout segmented EPI ex-vivo scans with b= 0 s/mm^2 were reconstructed. In the non-optimized scan (left and middle columns), the amplitude, rather than the duration, of the readout gradient blip was varied between shots, and the re-gridding algorithm for shot-dependent readout positions was not unoptimized. This can cause substantial shot-dependent phase variations due to system imperfections (e.g., eddy currents), resulting in visible artifacts near sharp edges ("local ghosts", left column) as a mixed effect with a sharp point-spread-function (for non-central k-space segments), if multiplying the complex conjugate phase maps in an image-space correction. Note that, these non-physiological phase fluctuations are also included by the kernel-estimated phase maps. Applying a Kaiser window (Kaiser coefficient = 3.0) to each readout segment before phase map multiplication substantially reduces these local ghosts artifacts (middle column). The k-space MTF due to Kaiser filters is compensated by an inverse filter. In this way, the multi-shot artifacts due to shot-dependent eddy currents could be properly monitored and removed by a few signal processing steps.

In a reference scan with optimized readout blips and re-gridding (right column), the trapezoidal waveform duration was adjusted while maintaining the amplitude and gradient ramp nearly consistent. The residue artifacts around the sharp edge are eliminated, which is verified to be caused by shot-dependent phase variations associated with system imperfections (since ex-vivo phantom has minimal motion).

The bottom row shows that the removal of shot-dependent physiological phase may also benefit from low-resolution apodization, if using a phase correction approach with multiplication of complex

conjugate phase maps in image-space. Similar to the upper row, the sharp point-spread-function may affect the direct multiplication of conjugate phase maps onto the partial image. Here, applying Kaiser filter before phase maps multiplication (middle column) resolves finer texture details compared to the unfiltered image (left column). The MTF caused by k-space apodization and its corresponding inverse filter is shown on the right.

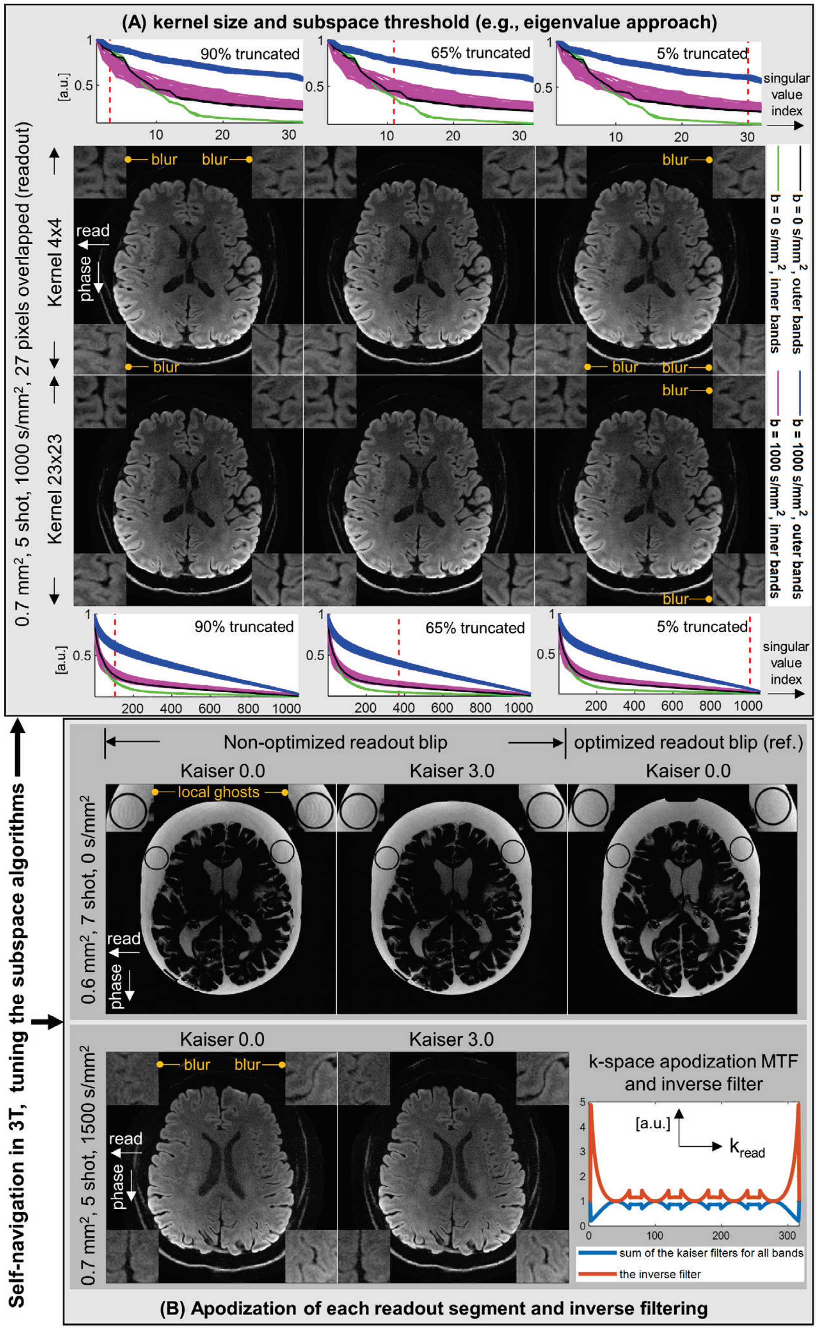

Figure S6. Reconstruction quality with respect to kernel size, subspace threshold and low-resolution apodization. (A) impact of kernel size and subspace threshold. (B) Effects of apodization on each EPI segment and the corresponding inverse filter. The ex-vivo phantom had no physiological phase fluctuations, and can only suffer from shot-dependent system imperfections. The diffusion-weighted images are shown as the sum image for a total of 20 diffusion directions.

5.3 Diffusion phase maps and kernels with increasing b-values, to examine smoothness and kernel width.

Figure S7 shows the image-space phase maps and their Fourier transform as k-space kernels, from 5 repetitions of single-shot EPI with b-values 1000, 2000, and 3000 s/mm^2. The typical kernel window size or width mostly-used in this paper (i.e., 7x7) are indicated by red rectangles or lines. Note that, sharp truncation of image-space background can make the k-space kernel more spread than they actually are. As the b-value increases, the k-space kernel becomes more spread due to more abrupt image-space phase variations.

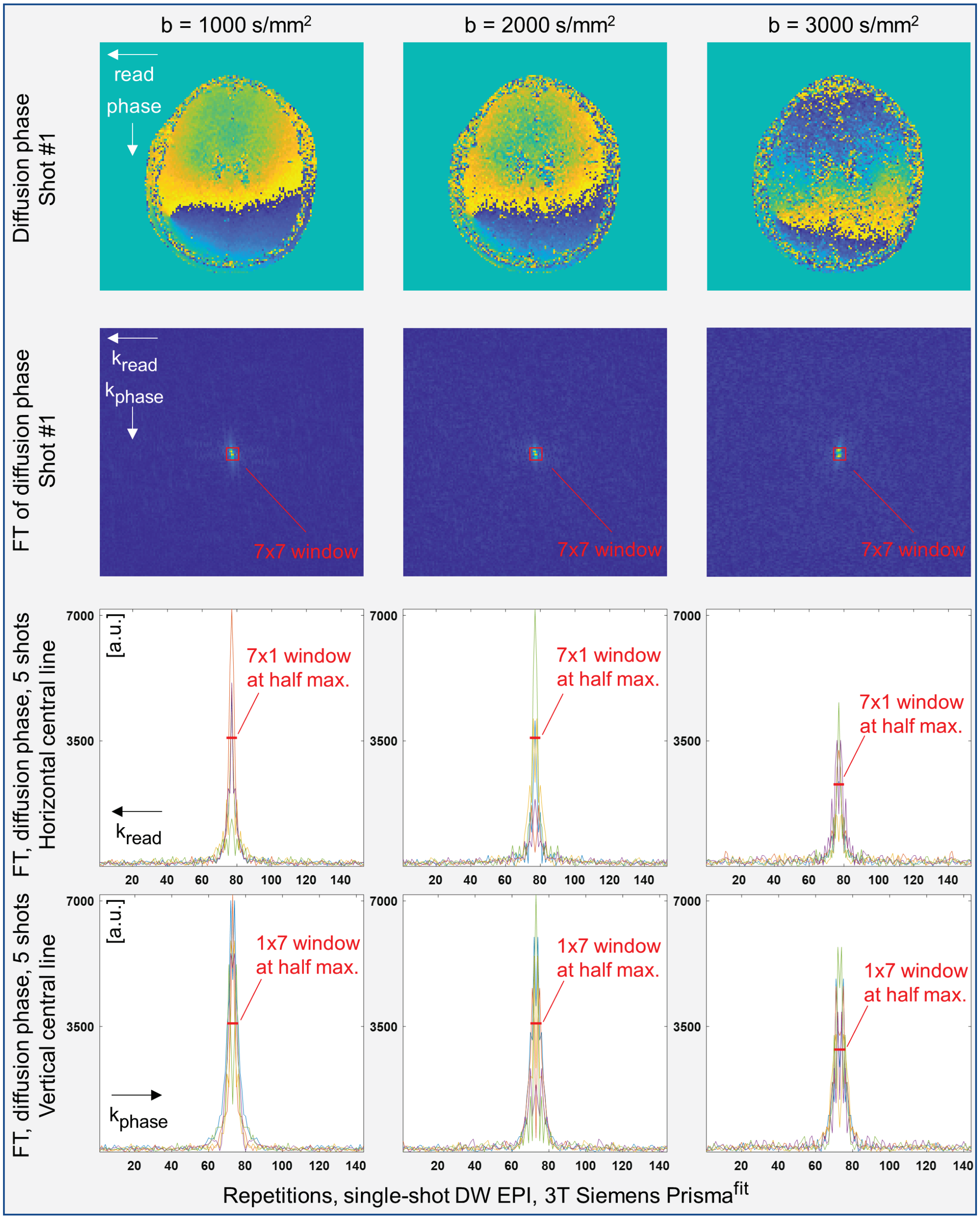

Figure S7. Diffusion phase fluctuations represented as image-space maps and k-space kernels, for b-values 1000, 2000, 3000 s/mm^2. Five repetitions (shot #1-5) of single-shot diffusion-weighted EPI were performed for different b-values. Row 1: The 2D image phase maps for shot#1. Row 2: The Fourier transform of image-space phase maps, as the diffusion phase kernels (absolute values displayed), for shot#1. A commonly used 7x7 kernel window (red rectangle) illustrates the portion of diffusion phase information captured by this kernel size across diffusion weighting. Row 3-4: Two 1D distribution of the k-space kernels are plotted to indicate the k-space range of

diffusion phases, with the kernel size marked at half of the maximum values. As b-value increases, the k-space diffusion kernels become more spread. Note that, due to sharp image-space truncation from background (finite object support), the k-space kernel distributions might appear more spread than they actually are.

6 Readout-segmented EPI scans with increasing diffusion-weighting

Figure S8 shows 3T readout-segmented EPI reconstructions with different diffusion-weighted b-values, comparing two approaches:

- Self-navigation with the eigenvalue approach (ESPIRiT-type operation)
- Corrections by 2D navigator with subspace extraction (here, also the eigenvalue approach).

For b = 0 s/mm^2, 500 s/mm^2, and 1000 s/mm^2, there is no noticeable difference in the resolution enhancement between these two approaches, except for a very subtle blur at one location in 7-shot rs-EPI ("slight blur", 7-shot, b = 1000 s/mm^2).

As the b-value increases to 1500 s/mm^2, the reconstructed images generally become noisier. In the 5-shot rs-EPI case, the reconstruction quality by the self-navigation and the 2D navigator with the subspace algorithm remain very similar. However, for 7-shot rs-EPI, the self-navigation method appears more blurred across the entire FOV compared to the navigator correction with subspace extraction, which can be possibly due to low-SNR in the most outer k-space overlapped bands.

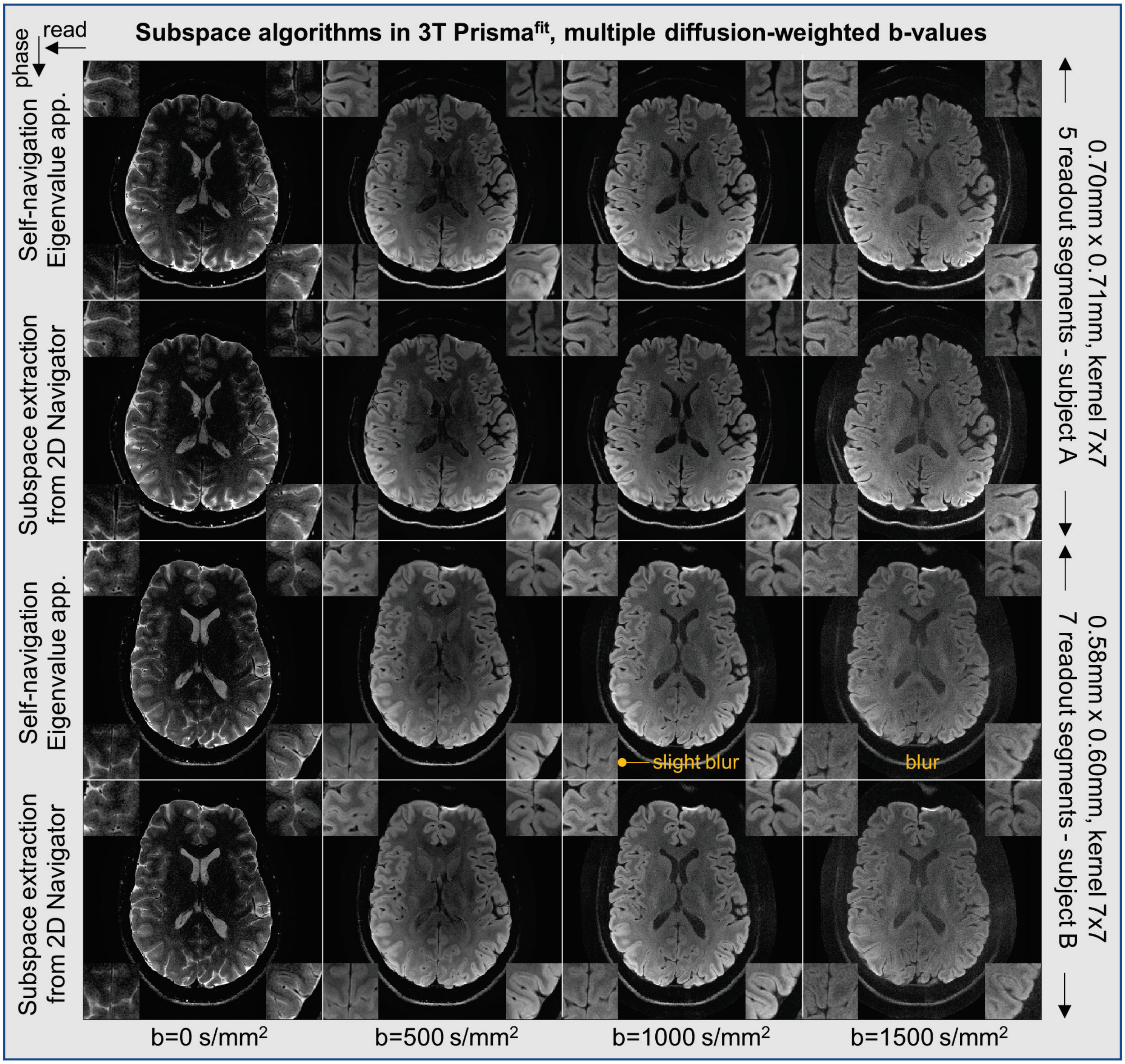

Figure S8. Readout-segmented EPI sequences with increasing diffusion-weighting, to visualize the SNR dependency on b-values ranging from 0 to 1500 s/mm^2. The diffusion-weighted images are shown as the sum images for a total of 20 diffusion directions.

7 More slice positions for low and high diffusion directions

Figure S9 shows the same dataset in manuscript's Figure 9 (32 diffusion directions, 2 averages) with more slice positions and uncorrected multi-shot reconstructions. Note, subtle artifacts are occasionally

found in certain slices (e.g., here, slice#3) using the 4-shot mosaic EPI. Such slice potentially has worse B_0 inhomogeneity, which requires further optimizations in (within-shot) trajectory and B_0 maps corrections.

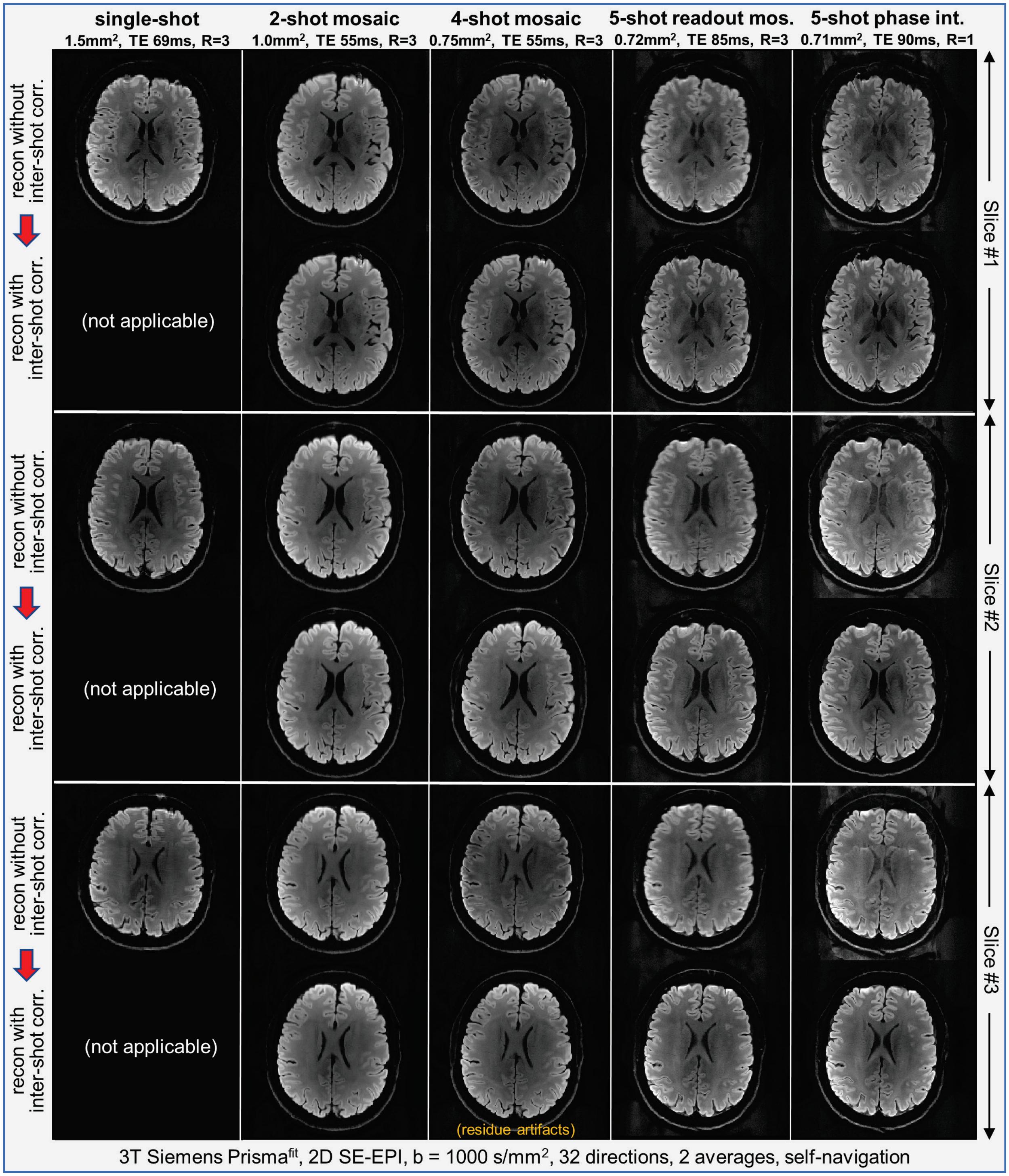

Figure S9. The same diffusion-weighted dataset (1000 s/mm^2, 32 diffusion directions, and 2 averages) in manuscript's Figure 9, with more slice positions, and uncorrected multi-shot reconstructions to reveal possible artifact patterns which may be helpful for protocol selections. The ESPs are the same as the corresponding sequences with identical trajectories in manuscript's Figure 5.

Figure S10-S13 show the same dataset in manuscript's Figure 5 (sum image with 3 diffusion directions, CV maps in 1 diffusion direction). Note, artifacts (blurring) are occasionally found in certain slices (here, Figure S10) using 4-shot mosaic EPI, which may require further optimized trajectory and B_0 maps corrections. Generally, self-navigation based on the proposed overlap-kernel EPI equal or better shot-to-shot phase calibration quality compared to 2D navigator corrections (although also using kernel extraction approaches), indicated by CV maps. The lower the CV values correspond to the higher time-stability. Note, additional artifacts or residue ghosts can be found in 2-shot mosaic and 5-shot phase-interleaved EPI, as well as conventional MUSE using image-space total variation phase estimations. These artifacts are minimized in the self-navigation approaches.

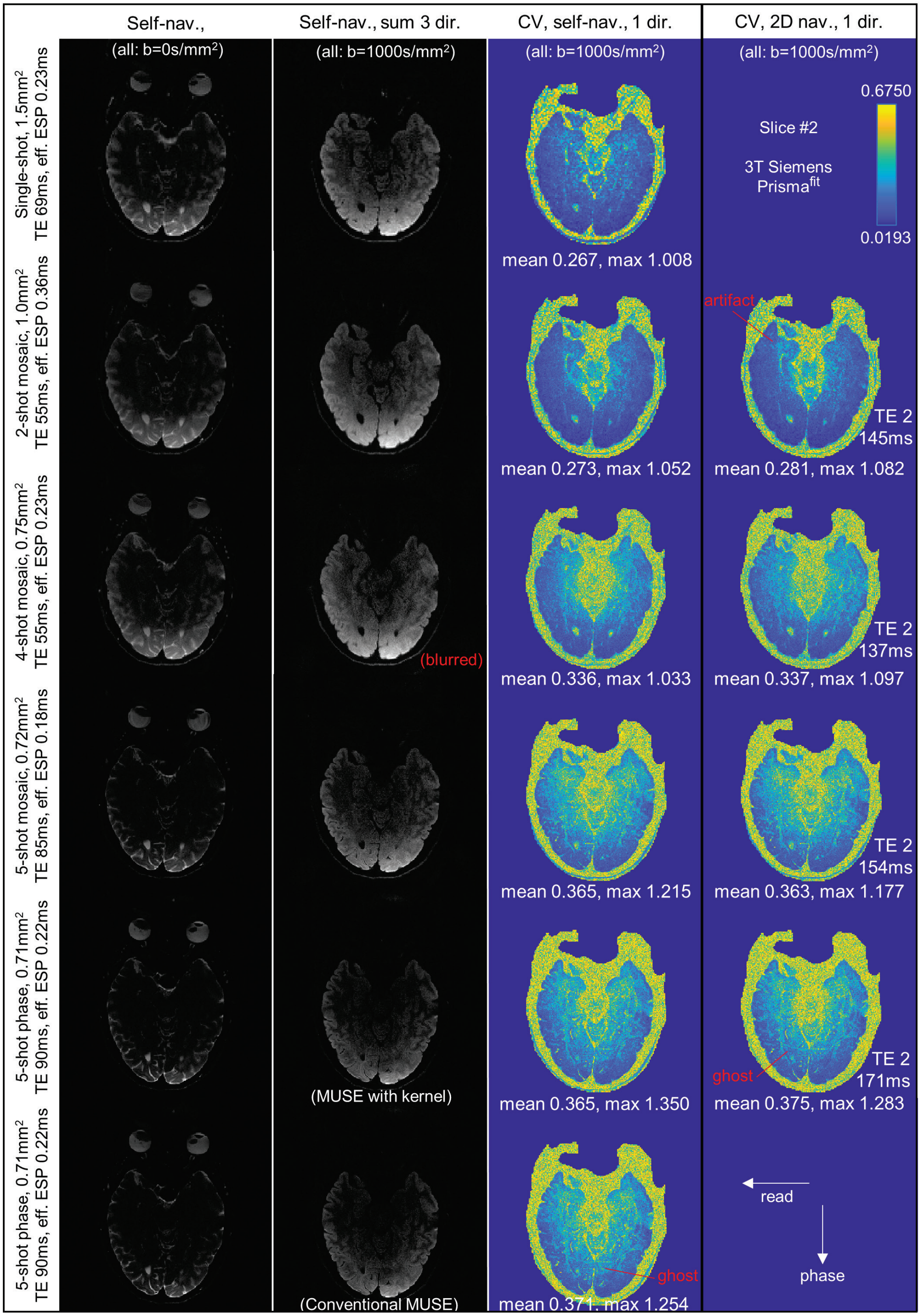

Figure S10. The same diffusion-weighted dataset in manuscript's Figure 5, with also non-diffusion-weighted images shown, in another slice position. The self-navigated sum images consisting of 3 diffusion directions, and

coefficient-of-variation maps for one diffusion direction over 10 repetitions are shown. In this slice with potentially stronger B_0 inhomogeneity, the 4-shot mosaic self-navigated EPI appears blurred, although its CV maps remain smaller, relative to 5-shot readout-segmented and phase-interleaved EPI. Similar to other slice positions, additional artifacts or residue ghosts can be found in the 2-shot mosaic and 5-shot phase-interleaved EPI with 2D navigator corrections, as well as the conventional MUSE. These are well-minimized in our overlap-kernel self-navigation techniques. Note, the effective ESP (not ESP) are shown, as the ESP divided by k-space undersampling (including possible phase-interleaved) factor in each shot.

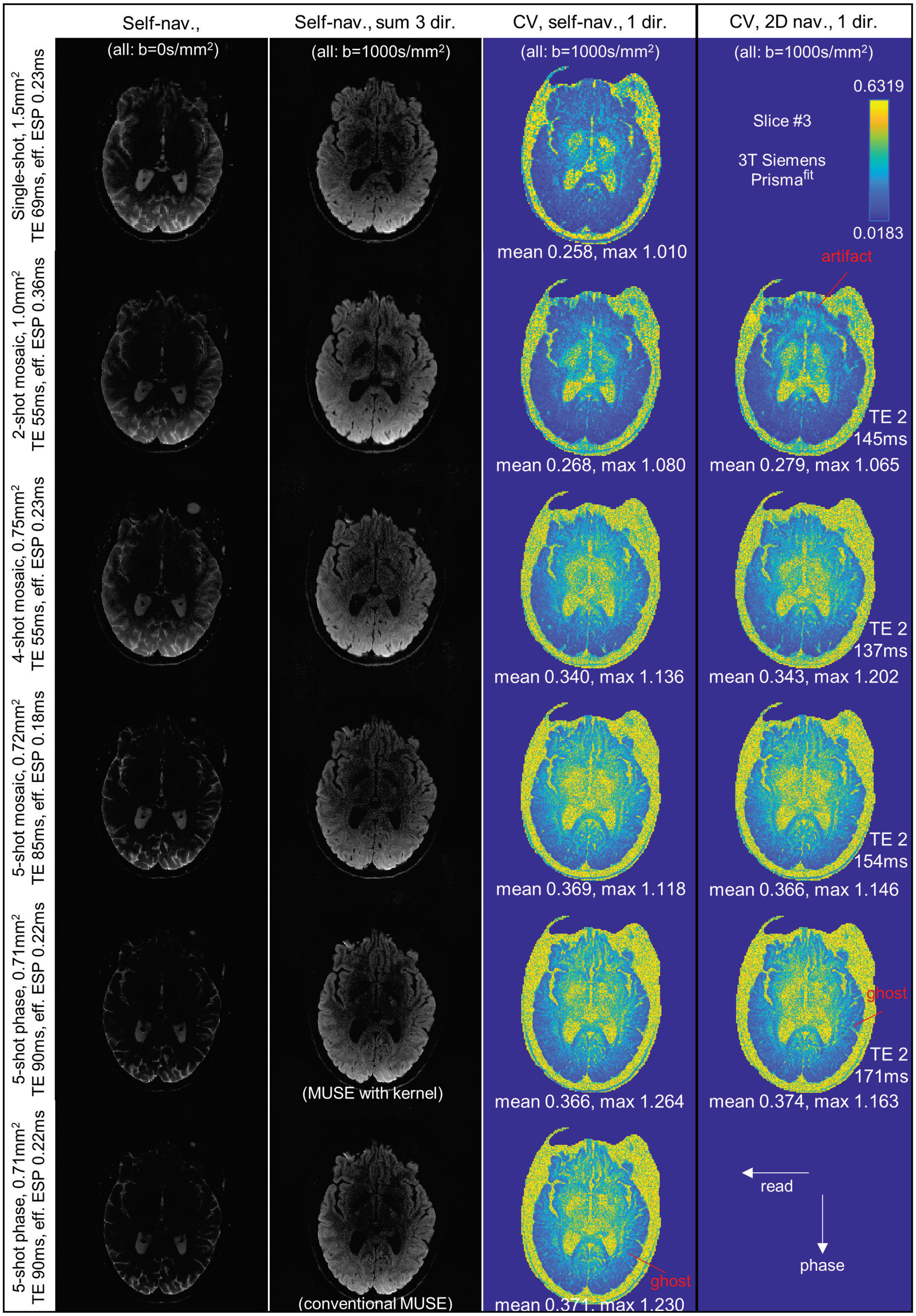

Self-nav.,
Self-nav., sum 3 dir.
CV, self-nav., 1 dir.
CV, 2D nav., 1 dir.
(all: b=0s/mm²)
(all: b=1000s/mm²)
(all: b=1000s/mm²)
(all: b=1000s/mm²)
0.6319
Slice #3
3T Siemens Prisma^fit
0.0183
Single-shot, 1.5mm² TE 69ms, eff. ESP 0.23ms
mean 0.258, max 1.010
artifact
2-shot mosaic, 1.0mm² TE 55ms, eff. ESP 0.36ms
TE 2 145ms
mean 0.268, max 1.080
mean 0.279, max 1.065
4-shot mosaic, 0.75mm² TE 55ms, eff. ESP 0.23ms
TE 2 137ms
mean 0.340, max 1.136
mean 0.343, max 1.202
5-shot mosaic, 0.72mm² TE 85ms, eff. ESP 0.18ms
TE 2 154ms
mean 0.369, max 1.118
mean 0.366, max 1.146
5-shot phase, 0.71mm² TE 90ms, eff. ESP 0.22ms
ghost
TE 2 171ms
(MUSE with kernel)
mean 0.366, max 1.264
mean 0.374, max 1.163
5-shot phase, 0.71mm² TE 90ms, eff. ESP 0.22ms
read
phase
ghost
(conventional MUSE)
mean 0.371, max 1.230

Figure S11. The same diffusion-weighted dataset in manuscript's Figure 5, with also non-diffusion-weighted images shown, in another slice position. The self-navigated sum images consisting of 3 diffusion directions, and coefficient-of-variation maps for one diffusion direction over 10 repetitions are shown. Similar to other slice positions, additional artifacts or residue ghosts can be found in the 2-shot mosaic and 5-shot phase-interleaved EPI with 2D navigator corrections, as well as the conventional MUSE. These are well-minimized in our overlap-kernel self-navigation techniques. Note, the effective ESP (not ESP) are shown, as the ESP divided by k-space undersampling (including possible phase-interleaved) factor in each shot.

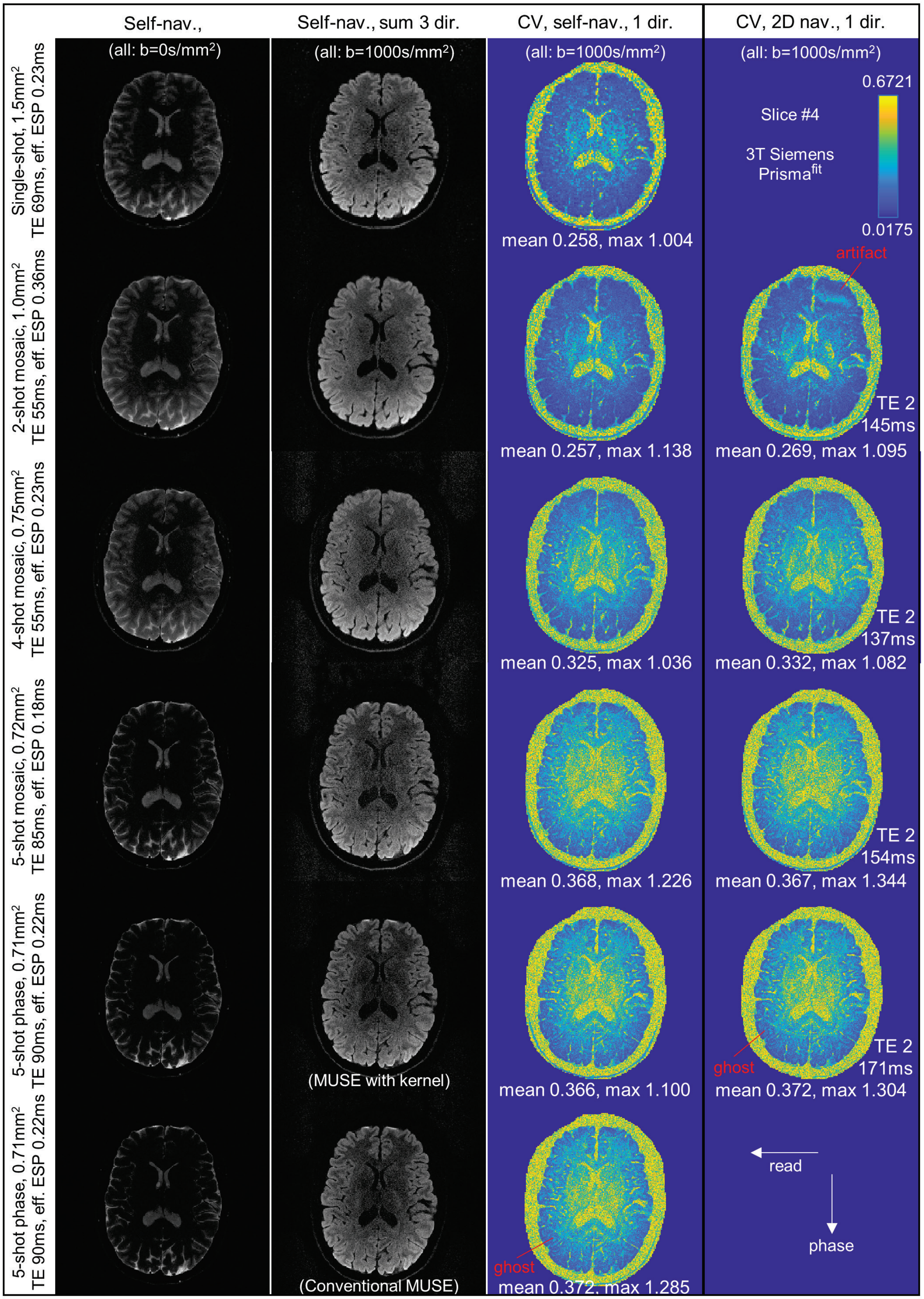

Self-nav.,
Self-nav., sum 3 dir.
CV, self-nav., 1 dir.
CV, 2D nav., 1 dir.
(all: b=0s/mm2)
(all: b=1000s/mm2)
(all: b=1000s/mm2)
(all: b=1000s/mm2)
Single-shot, 1.5mm2
TE 69ms, eff. ESP 0.23ms
2-shot mosaic, 1.0mm2
TE 55ms, eff. ESP 0.36ms
4-shot mosaic, 0.75mm2
TE 55ms, eff. ESP 0.23ms
5-shot mosaic, 0.72mm2
TE 85ms, eff. ESP 0.18ms
5-shot phase, 0.71mm2
TE 90ms, eff. ESP 0.22ms
5-shot phase, 0.71mm2
TE 90ms, eff. ESP 0.22ms
Slice #4
3T Siemens
Prismafit
0.6721
0.0175
mean 0.258, max 1.004
artifact
TE 2
145ms
mean 0.257, max 1.138
mean 0.269, max 1.095
TE 2
137ms
mean 0.325, max 1.036
mean 0.332, max 1.082
TE 2
154ms
mean 0.368, max 1.226
mean 0.367, max 1.344
(MUSE with kernel)
TE 2
171ms
ghost
mean 0.366, max 1.100
mean 0.372, max 1.304
read
phase
(Conventional MUSE)
ghost
mean 0.372, max 1.285

Figure S12. The same diffusion-weighted dataset in manuscript's Figure 5, with also non-diffusion-weighted images shown, in another slice position. The self-navigated sum images consisting of 3 diffusion directions, and coefficient-of-variation maps for one diffusion direction over 10 repetitions are shown. In this slice with potentially stronger B_0 inhomogeneity, the 4-shot mosaic self-navigated EPI appears blurred, although its CV maps remain smaller, relative to 5-shot readout-segmented and phase-interleaved EPI. Similar to other slice positions, additional artifacts or residue ghosts can be found in the 2-shot mosaic and 5-shot phase-interleaved EPI with 2D navigator corrections, as well as the conventional MUSE. These are well-minimized in our overlap-kernel self-navigation techniques. Note, the effective ESP (not ESP) are shown, as the ESP divided by k-space undersampling (including possible phase-interleaved) factor in each shot.

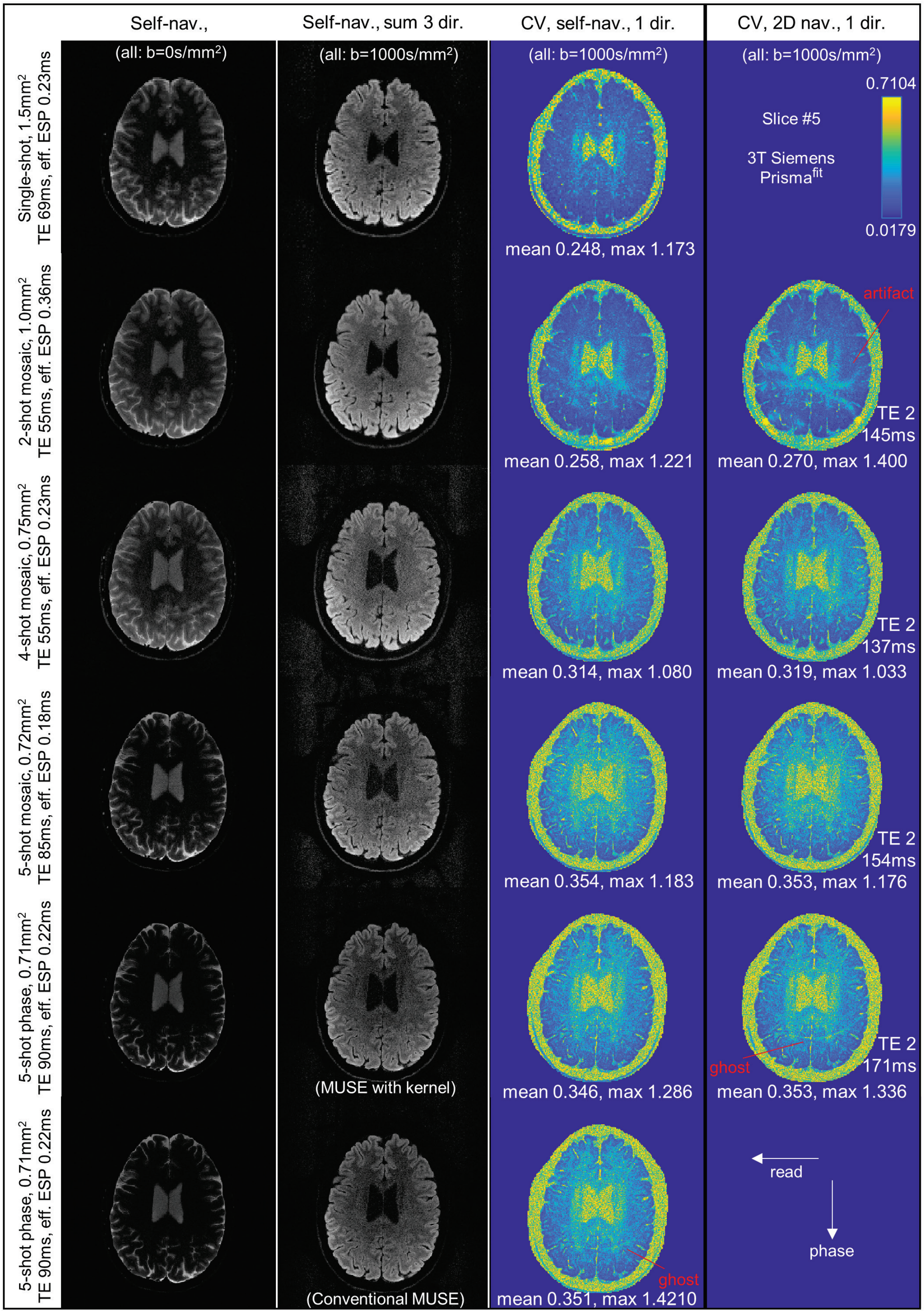
Self-nav.,
Self-nav., sum 3 dir.
CV, self-nav., 1 dir.
CV, 2D nav., 1 dir.
(all: b=0s/mm²)
(all: b=1000s/mm²)
(all: b=1000s/mm²)
(all: b=1000s/mm²)
Single-shot, 1.5mm²
TE 69ms, eff. ESP 0.23ms
2-shot mosaic, 1.0mm²
TE 55ms, eff. ESP 0.36ms
4-shot mosaic, 0.75mm²
TE 55ms, eff. ESP 0.23ms
5-shot mosaic, 0.72mm²
TE 85ms, eff. ESP 0.18ms
5-shot phase, 0.71mm²
TE 90ms, eff. ESP 0.22ms
5-shot phase, 0.71mm²
TE 90ms, eff. ESP 0.22ms
0.7104
0.0179
Slice #5
3T Siemens
Prisma^fit
mean 0.248, max 1.173
artifact
TE 2
145ms
mean 0.258, max 1.221
mean 0.270, max 1.400
TE 2
137ms
mean 0.314, max 1.080
mean 0.319, max 1.033
TE 2
154ms
mean 0.354, max 1.183
mean 0.353, max 1.176
TE 2
171ms
ghost
(MUSE with kernel)
mean 0.346, max 1.286
mean 0.353, max 1.336
read
phase
ghost
(Conventional MUSE)
mean 0.351, max 1.4210

Figure S13. The same diffusion-weighted dataset in manuscript's Figure 5, with also non-diffusion-weighted images shown, in another slice position. The self-navigated sum images consisting of 3 diffusion directions, and coefficient-of-variation maps for one diffusion direction over 10 repetitions are shown. In this slice with potentially stronger B_0 inhomogeneity, the 4-shot mosaic self-navigated EPI appears blurred, although its CV maps remain smaller, relative to 5-shot readout-segmented and phase-interleaved EPI. Similar to other slice positions, additional artifacts or residue ghosts can be found in the 2-shot mosaic and 5-shot phase-interleaved EPI with 2D navigator corrections, as well as the conventional MUSE. These are well-minimized in our overlap-kernel self-navigation techniques. Note, the effective ESP (not ESP) are shown, as the ESP divided by k-space undersampling (including possible phase-interleaved) factor in each shot.

8 Protocols

The sequence protocols in different scans are documented in details below.

8.1 General sequence parameters

Both imaging echoes and optional 2D navigator echoes with identical echo-spacing (ESP) and parallel imaging acceleration were obtained by separate spin-echo refocusing. The 2D pixel size is written as [mm along readout] x [mm along phase]. The readout gradient (RO) is in unit of (mT/m)/(T/m/s). $T_{1\,shot}$ is the time duration for a shot of acquisition including the optional 2D navigator scan. The diffusion directions (diff.) were calculated by Fibonacci sphere sampling. The overlap pixel number (overlap) corresponds to the actual overlapped k-space between shots, where phase-interleaved EPI always has an ACS region of 50x50 cropped from intermediate reconstructions of each shot. The parallel imaging acceleration factor (R) is the k-space undersampling factor considering all shots. The volumetric 1-shot TR (one shot for acquiring all slices) was 4s for SE-EPI, and 1s for GE-EPI.

8.2 General reconstruction parameters

The images were reconstructed offline in MATLAB (CPU, 2xAMD EPYC 7452, 2.35GHz, 64 cores, 2 hyper-threads/core). In the phase-interleaved EPI, data in shots were first SENSE-reconstructed with ESPIRiT map, where inter-shot phase maps were estimated from kernel extraction from a central 50x50 k-space region. All shots of data and maps were incorporated into a forward model to reconstruct a final image,

as the reconstruction approach two in the manuscript's section 2.4. For other sequences, data in shots were first SENSE-reconstructed with ESPIRiT RF maps, where inter-shot phase maps were then estimated from ESPIRiT-type operation with kernel size 7x7. The partial image in shots were multiplied by the complex conjugate of the corresponding phase maps, summed up, Fourier transformed into k-space, multiplied by an inverse filter, and Fourier transformed back to image-space, as reconstruction approach one in manuscript section 2.4. In 4-shot mosaic EPI, Kaiser apodization filtering (3.0) on each partial image was done to reduce ringing, and was removed by the inverse filter in a later step.

8.3 Figure 2(A)

15 slices were acquired with TE 85ms. The b-values were 0 s/mm^2 and 2000 s/mm^2. Resolution is 1.69 mm x 1.67 mm.

8.4 Figure 4, S2

EPI sequence protocols for Figure 4 and S2 are shown in Table S1. Three slices with 220mm^2 FOV, 3mm thickness and 4.5mm inter-slice distance were acquired. The overlap pixel for 4-mosaic is either 13x157 or 19x155, with the table value taking the minimum. Volumetric TR for all slices was 4s. In 2-shot mosaic, 2-shot phase-interleaved and 4-shot mosaic EPI, partial Fourier acquisition was disabled, while others had a partial Fourier factor 6/8.

In reconstruction using 2D navigators, the phase maps were replaced by ones extracted from 2D navigator data, with ESPIRiT-type operation using 7x7 kernel.

seq.	res.	b-value	TE	ESP	RO	$T_{1\,shot}$	diff.	overlap	R
2-mos.	1.0mm^2	3000s/mm^2	74ms/162ms	1.08ms	27/135	193ms	16	13x222	3
2-inter.	1.0mm^2	3000s/mm^2	98ms/148ms	1.09ms	27/136	180ms	16	50x50	3
4-mos.	0.75mm^2	2000s/mm^2	66ms/146ms	0.7ms	42/140	175ms	16	13x155	3
4-inter.	0.75mm^2	2000s/mm^2	84ms/137ms	1.1ms	42/139	169ms	16	50x50	2
5-rs.	0.72mm^2	1000s/mm^2	85ms/152ms	0.54ms	22/137	176ms	16	16x274	3

5-inter.	0.71mm^2	1000s/mm^2	90ms/170ms	1.1ms	43/138	214ms	16	50x50	1

Table S1. SE-EPI in Figure 4 and Figure S2.

8.5 Figure 5, S10-S13

The sequence protocols for EPI scans in Figure 5, Figure S10-S13 are shown in Table S2. The b-value is 1000 s/mm^2. Each sequence was performed with 10 repetitions.

SE seq.	res.	TE	ESP	RO	$T_{1\ shot}$	slice no.	overlap	R
1-shot	1.4mm x 1.5mm	69ms	0.69ms	42/140	105ms	7	/	3
2-mos.	1.0mm x 1.0mm	55ms/145ms	1.08ms	27/135	171ms	7	13x219	3
4-mos.	0.73mm x 0.75mm	55ms/137ms	0.7ms	42/140	160ms	7	13x157, 19x155	3
5-rs.	0.72mm x 0.71mm	85ms/154ms	0.54ms	22/137	176ms	7	16x274	3
5-inter.	0.68mm x 0.71mm	90ms/171ms	1.1ms	43/138	212ms	7	50x50	1

Table S2. SE-EPI in Figure 5, S10-S13.

8.6 Figure 6-7

The sequence protocols for readout-segmented EPI scans in Figure 6-7 are shown in Table S3. 6 slices were acquired with 3mm thickness, 4.5mm inter-slice distance.

The row 1 shows images with shot-to-shot phase fluctuations uncorrected.

The row 2 shows images with shot-to-shot phase fluctuations estimated by direct estimation of interpolation kernels (GRAPPA-type operation), with the kernel size 7x7.

The row 3 shows images with shot-to-shot phase fluctuations estimated by eigenvalue approach (ESPIRiT-type operation). The kernel size was empirically chosen as 4x4 for 3-rs EPI and 5-rs EPI, and 6x6 for 7-rs EPI.

The row 4 shows images with shot-to-shot phase fluctuations estimated by zero-filled 2D navigator. Namely, 2D navigator data were zero-filled in k-space, and smoothed by a filter (Kaiser 5.0), to extrapolate onto high-resolution grid for reconstruction. The reconstructed 2D navigator size was set to be 107x37, 78x37, 73x37 for 3-rs EPI, 5-rs EPI and 7-rs EPI, respectively.

The row 5 shows images with shot-to-shot phase fluctuations estimated by kernel extraction from 2D navigator. Namely, ESPIRiT-type operation was used to estimate shot-dependent phase fluctuations from 2D navigator data, with kernel size 7x7.

seq.	res.	b-value	TE	ESP	RO	$T_{1\ shot}$	diff.	overlap	R
3-rs.	0.8mm^2	1000s/mm^2	80ms/142ms	0.62ms	27/136	162ms	20	18x241	3
5-rs.	0.7mm^2	2000s/mm^2	87ms/152ms	0.52ms	25/138	171ms	20	18x274	3
7-rs.	0.6mm^2	3000s/mm^2	95ms/171ms	0.53ms	20/139	190ms	20	17x337	3

Table S3. Readout-segmented EPI in Figure 6-7.

8.7 Figure 8

The sequence protocols for 5-shot readout-segmented EPI scans in Figure 8 are shown in Table S4. In both long and short Diffusion gradient (i.e., denoted by DW grad. (mT/m)/(T/m/s)) scans, 5 slices with 3mm thickness and 4.5mm inter-slice distance were acquired. The inter-shot phase fluctuations were estimated by ESPIRiT-type operation for both self-navigation (kernel 8x8) and 2D navigator corrections (kernel 7x7).

DW grad.	res.	b-value	TE	ESP	RO	$T_{1\ shot}$	diff. dir.	overlap

43/164	0.72mm²	2000s/mm²	97ms/168ms	0.58ms	19/135	197ms	16	16x274
167/78	0.72mm²	2000s/mm²	76ms/147ms	0.58ms	19/135	176ms	16	16x274

Table S4. SE-EPI with long and short diffusion-weighted gradients in a 3T Siemens Cima.X scanner, as in Figure 8.

8.8 Figure 9, S9

The sequence protocols for self-navigated SE-EPI and GE-EPI scans in Figure 9 and S9 are shown in Table S5, S6. The slice thickness was 3mm with 4.5mm inter-slice distance. The kernel size for extracting phase fluctuations was 7x7 for all scans. The b-value for all SE-EPI diffusion scans was 1000 s/mm^2, with 32 diffusion directions. In 2-shot mosaic and 4-shot mosaic SE-EPI, the slice selection gradient polarity for RF excitation was reversed relative to the one for RF refocusing to reduce fat shift artifacts. The TE for all GE-EPI was 30ms.

SE seq.	res.	TE	ESP	RO	$T_{1\,shot}$	slice no.	overlap	R
1-shot	1.4mm x 1.5mm	69ms	0.69ms	42/140	104ms	26	/	3
2-mos.	1.0mm x 1.0mm	55ms	1.08ms	27/135	135ms	6	13x219	3
4-mos.	0.73mm x 0.75mm	55ms	0.7ms	42/140	109ms	6	13x157,<br>19x155	3
5-rs.	0.72mm x 0.71mm	85ms	0.54ms	22/137	134ms	26	16x274	3
5-inter.	0.68mm x 0.71mm	90ms	1.1ms	43/138	148ms	26	50x50	1

Table S5. Self-navigated SE-EPI in Figure 9, S9.

GE seq.	res.	ESP	RO	$T_{1\,shot}$	slice no.	overlap	R
1-shot	1.4mm x 1.5mm	0.69ms	42/140	65ms	11	/	3
2-mos.	1.0mm x 1.0mm	1.08ms	27/135	91ms	11	13x151	3
4-mos.	0.73mm x 0.75mm	0.7ms	42/140	88ms	11	min.13x155	3
3-rs.	0.73mm x 0.75mm	0.64ms	27/137	81ms	11	17x259	3
3-inter.	1.1mm x 1.1mm	1.04ms	26/134	85ms	11	50x50	1

Table S6. Self-navigated GE-EPI in Figure 9.

8.9 Figure S4

The sequence protocols for EPI scans in Figure S4 are shown in Table S7. Two and three slices were acquired for 2-shot mosaic and 6-shot phase-interleaved EPI, with 3mm thickness, 4.5mm inter-slice distance, and 220mm^2 FOV. The 2-shot mosaic data were shot-combined with the approach one based on multiplying phase maps with partial images and removed k-space MTF with inverse filter. The 6-shot phase-interleaved were shot-combined with the approach two based on incorporating all shots and maps into a forward model reconstruction. The eigenvalue approach (ESPIRiT-type operation) was used with kernel size 7x7.

seq.	res.	b-value	TE	ESP	RO	$T_{1\,shot}$	diff.	overlap	R
2-mos.	1.0mm^2	2000s/mm^2	66ms	1.08ms	27/135	141ms	3	13x221	3
6-inter.	0.71mm^2	2500s/mm^2	96ms	1.1ms	43/138	209ms	12	50x50	1

Table S7. EPI in Figure S4.

8.10 Figure S5

The sequence protocols for both GRE for B_0 mapping and non-diffusion-weighted EPI in Figure S5 are shown in Table S8, S9. Two slices with 220mm^2 FOV, 3mm thickness and 4.5mm inter-slice distance were acquired. The B_0 maps were extrapolated from two GRE data with distinct TE, by image-space smoothing (Kaiser filter 5.0), ESPIRiT-type operation with 6x6 kernel size, and ESPIRiT-type operation with 20x20 kernel size, respectively. They were later incorporated into the forward model reconstruction. In EPI, ESPIRiT-type operation with kernel size 7x7 was used to remove inter-shot phase fluctuations, including shot-dependent eddy currents in non-diffusion-weighted image. Reconstruction approach two in manuscript 2.4 was used to combine shots. Kaiser apodization filtering (3.0) on each partial image was applied to reduce ringing, and was removed by the inverse filter in a later step.

seq.	TE	TR	slice	resolution
GRE	8ms	20ms	2	0.73mm^2
GRE	11ms	20ms	2	0.73mm^2

Table S8. GRE in Figure S5.

seq.	res.	TE	ESP	RO	$T_{1\ shot}$	overlap	R
4-mosaic	$0.75mm^2$	66ms	0.7ms	42/140	175ms	13x157, 19x155	3

Table S9. EPI in Figure S5.

8.11 Figure S6A

SE-EPI with 6 slices were acquired with 4.5mm inter-slice distance, with protocols in Table S10.

seq.	res.	b-value	TE	ESP	RO	$T_{1\ shot}$	diff.	overlap	R
5-rs.	$0.70mm^2$	$0,1000s/mm^2$	88ms/155ms	0.54ms	27/137	174ms	20	27x274	3

Table S10. SE-EPI for tuning kernel, in Figure S6A.

8.12 Figure S6B

Multi-slice SE-EPI scans were acquired. In 5-shot readout-segmented EPI, diffusion direction was 20, with protocols in Table S11.

seq.	res.	b-value	TE	ESP	RO	$T_{1\ shot}$	overlap
7-rs.	$0.60mm^2$	$0\ s/mm^2$	95ms	0.5ms	21/147	/	10x313
7-rs. optimized.	$0.60mm^2$	$0\ s/mm^2$	95ms/171ms	0.53ms	20/139	/	17x322
5-rs	$0.70mm^2$	$1500\ s/mm^2$	87ms/152ms	0.52ms	25/138	171ms	18x274

Table S11. SE-EPI for evaluating filter and inverse filter, in Figure S6B.

8.13 Figure S7

The sequence protocols for single-shot EPI with 5-shot repetitions are shown in Table S12. The b-values in different sequences are 1000, 2000, 3000 s/mm^2, respectively.

SE seq.	res.	TE	ESP	RO	$T_{1\,shot}$	slice no.	overlap	R
1-shot	1.4mm x 1.5mm	69ms	0.69ms	42/140	105ms	7	/	3
1-shot	1.4mm x 1.5mm	80ms	0.69ms	42/140	116ms	7	/	3
1-shot	1.4mm x 1.5mm	88ms	0.69ms	42/140	124ms	7	/	3

Table S12. SE-EPI in Figure S7.

8.14 Figure S8

Multi-slice SE-EPI scans were acquired, with b-values 0, 500, 1000, 1500 s/mm^2. The sequence timing in non-diffusion-weighted scan was identical to the scan with b=500 s/mm^2, but with the diffusion gradients disabled. The protocols are in Table S13.

seq.	res.	b-value	TE	ESP	RO	$T_{1\,shot}$	diff.	overlap	R
5-rs.	0.70mm^2	0s/mm^2	74ms/139ms	0.52ms	25/138	158ms	/	18x274	3
5-rs.	0.70mm^2	500s/mm^2	74ms/139ms	0.52ms	25/138	158ms	20	18x274	3
5-rs.	0.70mm^2	1000s/mm^2	81ms/146ms	0.52ms	25/138	165ms	20	18x274	3
5-rs.	0.70mm^2	1500s/mm^2	87ms/152ms	0.52ms	25/138	171ms	20	18x274	3
7-rs.	0.60mm^2	0s/mm^2	81ms/156ms	0.52ms	21/137	175ms	/	19x322	3
7-rs.	0.60mm^2	500s/mm^2	81ms/156ms	0.52ms	21/137	175ms	20	19x322	3
7-rs.	0.60mm^2	1000s/mm^2	88ms/163ms	0.52ms	21/137	182ms	20	19x322	3
7-rs.	0.60mm^2	1500s/mm^2	95ms/171ms	0.53ms	20/139	190ms	20	17x322	3

Table S13. SE-EPI for evaluating reconstruction with increasing diffusion-weighting, in Figure S8.

9 Implementation algorithms for shot-to-shot phase estimations

This section describes the implementation steps for different approaches to estimate shot-to-shot phase fluctuation maps from imaging or navigator data.

9.1 Direct estimation of interpolation kernels (GRAPPA-type operation)

1) The overlapped region by two consecutive shots (e.g. shot #n and #n+1) is selected as the calibration region, from which a calibration matrix is constructed using the sliding window.
2) Based on the calibration matrix, establish a linear relationship that interpolates a neighborhood of k-space data from shot #n to a data point from shot #n+1 (or vice versa). Note, only half columns in the calibration matrix in Figure 2D are needed for one directional interpolation.
3) Solve the system of linear equations describing the interpolation relationship as Eq. (3), to obtain the interpolation kernels. Here, we used Moore-Penrose inverse (e.g. “pinv” in MATLAB).

9.2 Eigenvalue approach (ESPIRiT-type operation)

1) The overlapped region by two consecutive shots (e.g. shot #n and #n+1) is selected as the calibration region, from which a calibration matrix is constructed using the sliding window.
2) Perform SVD on the calibration matrix A.
3) Discard the singular-vectors with small singular-value (noise space $V_{\perp}$).
4) Take the signal space singular-vectors $V_{\parallel}$, reshape into k-space filters, zero-filled and (inverse) Fourier transform to image-space.
5) At each image-space pixel q, perform SVD on the matrix G_q^H (dim.: shot, eigenvector index), obtain the primary left singular-vector and fill into the initialized phase fluctuation maps at the corresponding pixel location. Iterate over all pixels to produce the relative signal fluctuation maps for shot #n and #n+1. Take the phase of these signal fluctuation maps.

9.3 Zero-filled 2D navigator images

1) The 2D navigator images are SENSE reconstructed, Fourier transformed into k-space, low-pass filtered (e.g. Kaiser window 5.0), and zero-filled onto a high-resolution grid matching the final reconstructed image.

9.4 Kernel extraction from 2D navigator k-space data

1) The phase fluctuation maps are extracted from 2D navigator data using the kernel extraction subspace algorithms in the sections 9.1 -9.2 above. Namely, the 2D navigator k-space data in shots are used as the ACS regions.
2) The kernel extraction subspace algorithms can be described as a subspace “filter” to extract shot-to-shot phase maps from image texture.
